# Tarsus length as a simple and robust candidate for early sex determination in partridges across contrasting growing contexts: a case study in Rock partridge (*Alectoris graeca Meisner, 1804*)

**DOI:** 10.1016/j.vas.2026.100657

**Published:** 2026-04-12

**Authors:** Paolo Viola, Pedro Girotti, Pier Paolo Danieli, Marco Zaccaroni, Bruno Ronchi, Nadia Piscopo, Livia Lucentini, Riccardo Primi

**Affiliations:** a Department of Agriculture and Forestry Science, DAFNE, University of Tuscia, 01100 Viterbo, Italy; b Department of Biology, University of Florence, Via Madonna del Piano 6, 59100 Sesto Fiorentino, Italy; c Department of Veterinary Medicine and Animal Production, University of Naples Federico II, 80137 Naples, Italy; d Department of Chemistry, Biology and Biotechnology, University of Perugia, 06123 Perugia, Italy

**Keywords:** Discriminant analysis, Morphometrics, Captive breeding, Rearing conditions, Juvenile sexing, Reintroduction

## Abstract

Captive breeding of wild Galliform birds for restocking and reintroduction purposes requires efficient management strategies to optimise animal handling, welfare, and release outcomes. Among these, the early definition of sex ratio represents a relevant operational constraint, as sexual dimorphism in some Galliformes generally becomes evident only at advanced developmental stages. This study evaluated the applicability of simple morphometric measurements for early sex discrimination (< 56 days post-hatching) in juveniles of a partridge species (*Alectoris graeca*) reared under contrasting production systems (intensive vs. semi-natural). A total of 33 chicks were included. Body weight and linear body measurements were systematically recorded, and multivariate discriminant analysis was used to identify the most informative traits. Live weight, tarsus length, and head length emerged as the main predictors of sex. The most efficient and easily transferable discriminant model was obtained at 42 days post-hatching using tarsus length as the sole variable, achieving a high canonical correlation (0.89) and 100% classification accuracy under the present experimental conditions, regardless of the rearing system. The proposed approach identifies a practical, rapid, and low-cost candidate for early sexing of juveniles, showing consistent performance across rearing conditions and offering direct applications for breeding management, production planning, and veterinary supervision of captive-reared birds intended for release.

## Highlights

• Three body traits of partridges showed early and marked differences between sexes irrespective of the growth environment.
• Quick, cheap, and robust models for sex discrimination of juvenile partridges at 28 and 42 days have been described.
• Sex can be established as early as 42 days post hatching by measuring only the tarsus length.

## Introduction

Captive breeding of wild Galliform birds for restocking and reintroduction represents a specialized form of animal production that requires careful planning of breeding cycles, animal handling, and release schedules (IUCN/SSC, 2013; Collar et al., 2020). In addition to genetic background and provenance, which are key determinants of local adaptation and long-term population viability (Barilani et al., 2007; Brustenga et al., 2022; Fontaneto et al., 2022), and sanitary status (IUCN/SSC, 2013; Beckmann et al., 2022), the age at which juveniles are transferred or released plays a pivotal role in post-release performance. Early life stages are characterised by higher morphological and behavioural plasticity, potentially enhancing adaptability to novel environments (Whiteside et al., 2016; Tetzlaff et al., 2019).

From a management perspective, the definition of an appropriate sex ratio represents a key operational requirement, influencing breeding efficiency, production planning, and the demographic structure of released cohorts (Lambertucci et al., 2013; Guaricci et al., 2025). In many Galliform species, however, sexual dimorphism is weak or absent during juvenile development (Bernard-Laurent et al., 2003), limiting the feasibility of early sex determination. While both molecular techniques (Vucicevic et al., 2013; Guaricci et al., 2025) and morphometric approaches based on multiple traits have been proposed (Bernard-Laurent et al., 2003; Pépin, 1985; Weaver & Haskell, 1968; Woodard et al., 1985), their routine application in captive breeding is often constrained by costs, time requirements or operational complexity, particularly when large cohorts must be processed rapidly. Morphometric methods may rely on traits that are difficult to measure reliably in live birds, are influenced by captivity-related wear, or provide accurate discrimination only at later developmental stages, whereas molecular sexing, despite being reliable and minimally invasive (Vucicevic et al., 2013; Gandolfi et al., 2016), frequently fails to meet the requirements of speed and cost-effectiveness in day-to-day breeding management.

**Fig. 1:**
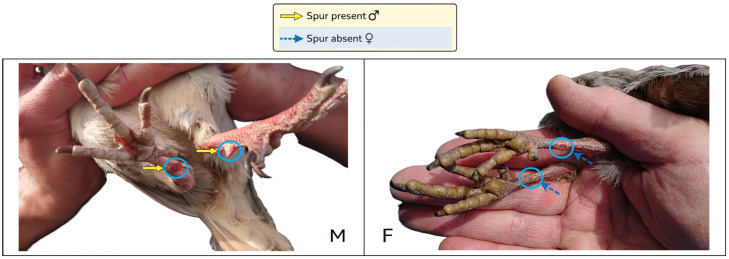
**Detail of metatarsal spurs.** Male (left) with well-developed metatarsal spur, and female (right) lacking spurs.

Partridges of the genus *Alectoris* are sexually monomorphic in plumage and develop reliable sex-discriminant traits only at advanced stages of growth or at sexual maturity (Bernard-Laurent et al., 2003) making early sex determination in juveniles particularly challenging. In adult, sex can be reliably determined by the presence of metatarsal spurs (Fig. 1), which are typically present in males and absent or rarely developed in females (Bernard-Laurent et al., 2017).

Within this genus, the Rock partridge (*Alectoris graeca Meisner, 1804*) represents a particularly informative study system, as it combines pronounced juvenile monomorphism with high conservation relevance and a long history of both in situ and ex situ management (Mihajlov et al., 2018; Viola et al., 2019; Zaccaroni et al., 2007). The species is the only continental *Alectoris* listed in both Annex I and Annex II/A of the EU Birds Directive, reflecting its importance for conservation-oriented breeding programs as well as for regulated game management.

In fact, according to the IUCN, the Rock partridge is classified as Near Threatened in Europe (BirdLife International, 2023) and as Vulnerable in Italy (Rondinini et al., 2022), where it occurs in three geographically distinct populations that should be managed as independent management units (sensu Moritz, 1994). These populations are affected by multiple pressures, including habitat fragmentation, introgression with released congeneric species, disturbance, poaching, unsustainable hunting, extreme climatic events, climate change and pathogen transmission between captive and wild populations (Barilani et al., 2007; Brusaferro et al., 2019; Budinski et al., 2010; Canonne et al., 2023; Cattadori et al., 2003; Fanelli et al., 2020; Fontaneto et al., 2022; Knaus et al., 2018; Pizzirani et al., 2020; Randi, 2008; Rippa et al., 2011; Trocchi et al., 2016). Within this context, national management strategies include the reinforcement of extant populations through restocking or reintroduction when considered appropriate (Trocchi et al., 2016).

Rearing conditions strongly influence morphological, physiological, and behavioural development in Galliform birds. Intensive systems may induce phenotypic alterations that negatively affect post-release performance, whereas semi-natural or enriched environments better support functional development but require greater management effort. Evidence from ex situ conservation studies indicates that morphology and behaviour shaped during early developmental stages influence survival after release (Viola et al., 2019; Zaccaroni et al., 2007), and that semi-natural rearing can improve reintroduction outcomes (Mihajlov et al., 2018). In Rock partridge juveniles reared under intensive conditions, significant morphological differences emerge from approximately 70 days post-hatching compared with birds raised in more complex environments (Viola et al., 2019), suggesting that transfer to acclimatization or release sites should occur earlier, preferably within the first eight weeks of life (≤ 56 days post-hatching), as also reported for the grey partridge (*Perdix perdix* Linnaeus*, 1758*) (Buner & Aebischer, 2008; Buner & Schaub, 2008).

Within this limited developmental window, restocking and reintroduction must be planned as quali-quantitative actions that account for production output, carrying capacity and target sex ratio (Lambertucci et al., 2013; Guaricci et al., 2025), making early, rapid, and cost-effective sex determination a relevant management tool.

The aim of this study was to develop a rapid, inexpensive, and operationally feasible approach for early sex determination in juvenile partridges under captive breeding conditions. Using the Rock partridge as a model species, we focused on identifying simple morphometric traits with early sex-discriminatory potential and evaluating their robustness across contrasting rearing conditions. Rather than proposing a universally applicable model, our objective was to define a generalizable analytical framework and to identify robust candidate traits that can be readily measured and subsequently calibrated within specific management, conservation, and breeding contexts, across different genetic backgrounds.

## Methods

### Animals and data collection

A wild Rock partridge breeding pair was used to produce, under ex situ conditions, two experimental rearing groups of related offspring at a farm in central Italy routinely engaged in partridge production (Viola et al., 2019). Genetic characterisation assigned the founders to the nominal subspecies (*A. g. graeca*), haplotype 10 (H10) (Brustenga et al., 2022; Randi et al., 2003), and excluded introgression with congeneric species.

The use of a single wild breeding pair was a deliberate methodological choice. Additional wild parental pairs were not available, and the inclusion of multigenerational captive individuals was avoided because captivity-driven genetic and phenotypic divergence is known to affect morphology and could have introduced a stronger and less controllable bias than reduced genetic variability (O’Regan & Kitchener, 2005; Viola et al., 2019). Under these constraints, controlling genetic background through a single wild pair was considered the most conservative approach for isolating the effects of contrasting rearing environments.

Assuming an approximately 1:1 sex ratio at hatching, chicks were randomly assigned to two experimental rearing groups: an intensive system (G1) and a wild-like system (G2). Sex was confirmed at sexual maturity based on the presence of tarsometatarsal spurs and by cloacal examination. Sixteen chicks (8 males and 8 females) were allocated to G1 and reared under the standard intensive conditions adopted by the farm, consisting of an initial indoor phase up to 35 days, followed by an indoor–outdoor phase with progressive expansion of the available open-air surface. A further 14 chicks (8 males and 6 females) were assigned to G2 and reared in a complex wild-like environment under the guidance of their natural parents throughout the study period.

The sample size was restricted to first-generation offspring of a single wild pair to ensure that all individuals were unequivocally attributable to pure Rock partridge already in captivity, thereby excluding potential bias associated with morphological changes known to affect multigenerational captive animals (O’Regan & Kitchener, 2005; Viola et al., 2019). Detailed information on rearing group composition, management strategies, experimental schedule, and data recording procedures is reported in Viola et al. (2019).

Birds were progressively funnelled from the enclosures into a small mesh corral through a system of low netted corridors (ca. 50 cm high), allowing controlled and low-stress capture. Individuals were then manually captured and gently restrained for measurements. All morphometric data were collected by a single operator to minimise inter-observer variability. Handling time was minimised, generally not exceeding one minute per individual, and birds were released immediately after processing.

**Fig. 2:**
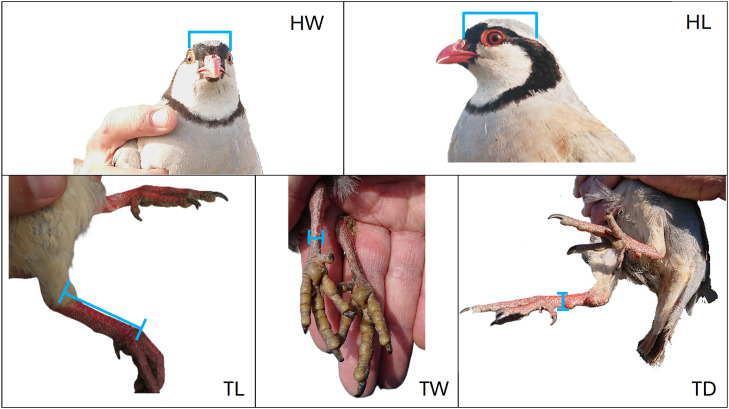
**Morphometric measurements recorded in this study.** HW: head width, measured between the orbital faces of the frontal bone; HL: head length, measured from the occipital region to the frontal bone; TL: tarsus length, measured from the distal end of the tarsometatarsus to the posterior condyle of the distal tibiotarsus; TD: tarsus depth and TW: tarsus width, measured at the distal margin of the spur scale as the antero-posterior thickness and medio-lateral width of the tarsometatarsus, respectively.

Six morphometric measurements were recorded fortnightly following standard procedures (De Beer et al., 2001; Çağlayan et al., 2011; Vucicevic et al., 2013; Pis, 2012; Viola et al., 2019). Live weight (LW) was measured using an electronic balance (precision: ±0.01 g) by placing each bird individually in a lightweight bag previously tared on the balance. The length of the tarsometatarsus was measured as the distance between its distal end and the posterior condyle of the distal end of the tibiotarsus; hereafter, this measurement is referred to as tarsus length (TL). Tarsus depth (TD) and tarsus width (TW) have been measured on the tarsometatarsus, at the level of the distal margin of the spur scale, as the antero-posterior thickness and the medio-lateral width of the tarsometatarsus, respectively. Head length (HL) was measured from the midpoint of the occipital region to the nasal face of the frontal bone, and head width (HW) was measured between the orbital faces of the frontal bone (Fig. 2). All linear measures were taken to the nearest 0.01 mm with a digital calliper (Mitutoyo Digital Caliper 500–181–30 mm 150, Digital step: 0.01 mm, Accuracy: ±0,02 mm).

### Data analysis

The analytical approach was designed to identify simple and transferable morphometric predictors of sex.

The analytical workflow was structured in three sequential steps aimed at identifying, refining, and validating discriminant models based on morphometric traits ensuring consistent discrimination across contexts.

Linear Discriminant Analysis (LDA) was performed using the MASS package (Venables & Ripley, 2002) in R (R Core Team, 2024). Prior to analysis, equality of covariance matrices was assessed using Box’s M test. A conservative significance threshold of 1 % (*p* = 0.01) was adopted throughout.

LDA was implemented using a stepwise procedure, assuming equal a priori probabilities and adopting the Mahalanobis distance as the variable selection criterion (Leotta et al., 2004). The discriminant intercept was calculated as the midpoint between sex-specific group centroids, and the sign of each discriminant function was oriented so that values of *D* > 0 corresponded to female classification. Bootstrap resampling (1000 iterations) was used to obtain robust estimates of classification performance. Discriminant function strength was assessed using canonical correlation (r) and Wilks’ Lambda (Wilks’). Internal validation was performed using the Jackknife leave-one-out procedure (Manly, 1994), whereby each observation is classified using a function derived from all remaining cases. Model performance was subsequently evaluated on an external sample, as described below.

To identify discriminant functions with the highest potential for transferability, a stepwise analytical strategy was adopted. Given the controlled genetic background of the study design, the terms external validation and external sample are hereafter used exclusively to indicate model transferability across contrasting rearing environments.

In the first step (model development), morphometric data collected from G1 were analysed using LDA, and the resulting discriminant functions were tested both on G1 (internal validation, equal a priori probability of 0.5) and on G2, which served as the external sample.

In the second step (trait selection), only body traits that were both robust to rearing conditions (i.e. minimally affected by environmental variation) and sufficiently discriminant to reliably distinguish individuals across contexts were retained, and potential improvements in sex classification accuracy were evaluated through internal (G1) and external (G2) validation. A morphological trait was considered robust when no significant differences between rearing groups were detected within each sex, and DRRS when significant differences between sexes were consistently detected across all sex × group combinations (males-G1 vs females-G1, males-G1 vs females-G2, males-G2 vs females-G1, males-G2 vs females-G2). Independent-sample *t*-tests were used for these comparisons, following verification of variance homogeneity with F-tests.

Finally (pooled model validation), data related to robust and DRRS traits from both rearing groups were pooled to incorporate the full range of phenotypic variability observed across systems. Seventy percent of the cases for each sex within each rearing group were randomly selected using the random number generator in Microsoft® Excel 365 and assembled into a pooled training dataset for LDA. The remaining 30 % of cases were retained as an independent test dataset for final external validation.

## Results and discussion

Since the main aim of this study was to provide morphometric-based sex determination models capable of effectively supporting restocking or reintroduction actions, sex discrimination was investigated from 28 to 42 days post-hatching (DPH). This time window was selected because it is functionally relevant for preparing groups at an appropriate developmental stage for transfer to acclimatisation or fostering sites and subsequent release within the optimal time limit (Buner & Aebischer, 2008; Buner & Schaub, 2008).

Discriminant analyses performed on all morphometric traits recorded at 28 and 42 DPH in the intensive rearing group (G1) retained tarsus length (TL) and live weight (LW) as discriminant variables at 28 DPH, and TL and head length (HL) at 42 DPH (Table 1 and Supplementary Material). At 28 DPH, the discriminant function correctly classified 100 % of females and 87.5 % of males in G1. However, its performance declined when applied to the wild-like group (G2), where three out of eight males (37.5 %) were incorrectly assigned to the female class. Bootstrap resampling (1000 iterations) confirmed high stability of the training performance, with a mean correct classification rate of 97.91 % (95 % CI: 87.5–100 %).

**Table 1:** Discriminant functions developed on the intensive rearing system (G1) and percentage of individuals in each rearing group correctly sexed at 28- and 42-days post hatching (DPH).

DPH	Discriminant function	Canonical r	Wilk’s Lambda	p-level	Males’ right assignment		Females’ right assignment		Bootstrap overall accuracy ( %)	
					G1	G2	G1	G2	G1 (train)	G2 (test)
28	*D*= - 0.636 (TL) - 0.056 (LW) + 27.77	0.99	0.02	<0.001	87.5 (8)	62.5 (8)	100 (8)	100 (6)	97.9 (87.5–100)	78.7 (57.1–92.7)
42	*D*= - 0.713 (TL) - 1.080 (HL) + 61.226	0.99	0.01	<0.001	100 (8)	100 (8)	87.5 (8)	100 (6)	98.7 (93.7–100)	100 (100–100)

TL: tarsus length (mm); HL: head length (mm); LW: live body weight (g); G1: intensive rearing condition; BS = Bootstrap sample (1000 iterations) G2: wild-like rearing condition. Values of *D* > 0 indicate female cases. The number of individuals in each sample is reported within brackets.

**Fig. 3:**
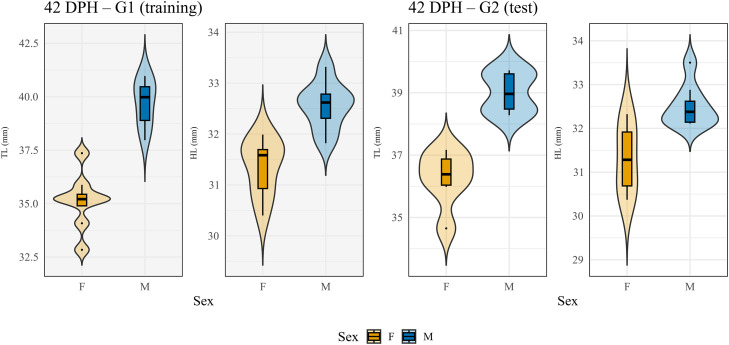
**Trait distribution by sex at 42 days post hatching (DPH) in the G1 training group (intensively reared) and the G2 external test group (wild-like reared).** Violin plots represent the distribution of tarsus length (TL) and head length (HL) for males and females, whit violin width proportional to the relative frequency of observations. Embedded boxplots show the median (central line), interquartile range (box), and data dispersion up to 1.5 times the interquartile range (whiskers); individual points indicate outliers when present. These traits were identified as robust and discriminant regardless of rearing conditions (DRRS) and were retained at 42 DPH in the discriminant analysis. F: female; M: male.

In contrast, the discriminant function developed at 42 DPH showed high accuracy in both rearing groups, with only one female misclassified during Jackknife validation in G1 and all individuals correctly classified in the external G2 sample (Fig. 3). Bootstrap resampling yielded a mean training accuracy of 98.74 % (95 % CI: 93.75–100 %), further supporting the robustness of this model.

Bernard-Laurent et al. (2003) previously assessed the exportability of morphometry-based discriminant models developed on hand-reared, genetically uncontrolled Alpine Rock partridges to wild populations, without success. These findings suggested the occurrence of environmentally driven morphological divergence among conspecifics exposed to contrasting conditions (Viola et al., 2019; Whiteside et al., 2016), supporting the hypothesis of limited model exportability across contexts. Contrary to these expectations, in the present study TL and HL allowed the development of an accurate and transferable sex discrimination function at 42 DPH.

**Fig. 4:**
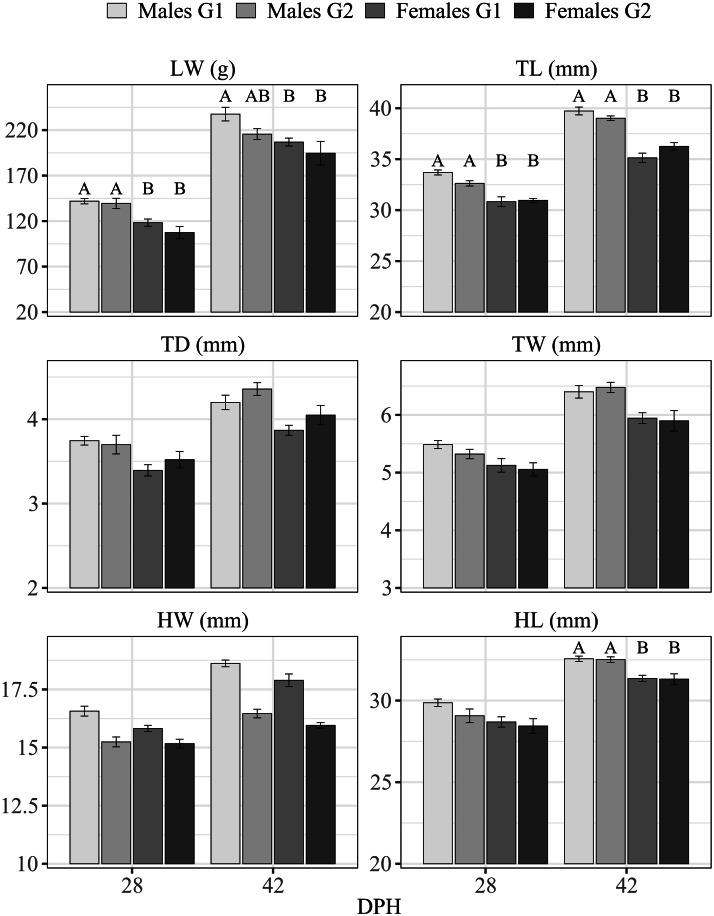
**Averages values (means ± SD) of each morphological trait detected at 28- and 42-days post hatching (DPH) on each sex within each rearing group.** LW: live body weight (g); TL: tarsus length (mm); TD: tarsus depth (mm); TW: tarsus width (mm); HW: head width (mm); HL: head length (mm); G1: intensive rearing condition; G2: wild-like rearing condition. ^A, B^*p* ≤ 0.01.

Analysis of differences between rearing groups across all sex × group combinations (Fig. 4) showed that at 28 DPH only LW and TL were simultaneously robust and discriminant regardless of rearing system (DRRS), whereas at 42 DPH both TL and HL met these criteria. Notably, at both 28 and 42 DPH, TL and HL values largely overlapped between rearing groups within each sex (Fig. 4), consistent with the findings of Bernard-Laurent et al. (2003), who reported no significant differences in TL between captive and wild A. g. saxatilis. This pattern may reflect the high heritability of tarsus length (Cava et al., 2019; Kondra & Shoffner, 1955). Furthermore, the present results are in agreement with Woodard et al. (1985), who detected significant sex-related differences in TL in *Alectoris chukar* chicks starting from 28 DPH, although reliable discrimination was achieved only after 10 weeks post-hatching, which is later than the optimal management window (≤ 56 DPH).

At 42 DPH, tarsus depth (TD) recorded in G2 exceeded mean values observed in G1, although the difference was not statistically significant (Fig. 4). This trend may reflect differences in locomotor activity and muscular development, as intensive systems tend to promote rapid early growth, whereas wild-like environments favour greater development of limb musculature (Santilli & Bagliacca, 2017; Whiteside et al., 2016).

Outliers in TL were observed in both groups but appeared more frequent in the intensively reared group (G1), where the reduced dispersion of values resulted in a tighter distribution around the median. In contrast, the broader interquartile range observed in the wild-like group (G2) indicates greater inter-individual variability, which reduces the prominence of extreme values within the distribution. The narrower spread in G1 likely reflects more homogeneous rearing conditions, including ad libitum resource availability, controlled temperature regimes, and the absence of environmental constraints. Conversely, the wider variation in G2 may be associated with greater environmental heterogeneity and variability in access to resources, leading to increased inter-individual differences and a less distinct emergence of outliers.

Notably, these outliers did not substantially affect model performance at 42 DPH, as classification accuracy remained unchanged in the pooled training and test datasets used for final model validation.

In the subsequent analytical step, only robust and DRRS morphometric traits were processed by discriminant analysis to assess potential improvements in sex classification accuracy. Notably, the stepwise procedure applied to the full set of morphometric traits in G1 had already selected TL and LW at 28 DPH and TL and HL at 42 DPH, exactly corresponding to the robust and DRRS traits identified in this second step (Fig. 3). This complete overlap indicates strong internal consistency in trait selection across analytical approaches. Consequently, the discriminant functions obtained in Step 2 were identical to those derived in Step 1, and classification accuracy remained unchanged in both internal (G1) and external (G2) validations (Table 1 and Supplementary Material).

The discriminant analysis performed on the pooled dataset identified the earliest significant sex discrimination at 28 DPH, retaining TL and LW as predictor variables (Table 2). This function correctly classified 75.0 % of males and 90.0 % of females in the training dataset, irrespective of rearing group, resulting in an overall Jackknife accuracy of 81.8 %. In the external test sample, overall accuracy reached 87.5 %, with all males (100 %) and 75.0 % of females correctly classified. Bootstrap resampling (1000 iterations) confirmed moderate robustness of this early-age model (mean accuracy = 92.15 %, 95 % CI: 81.78–100 %).

**Table 2:** Discriminant functions developed on robust and DRRS morphological traits of the pooled dataset (70 % of the cases of each sex of each rearing group) and percentage of individuals correctly sexed at 28- and 42-days post hatching (DPH).

DPH	Discriminant function	Canonical r	Wilk’s Lambda	p-level	Males’ right assignment		Females’ right assignment		Bootstrap overall accuracy ( %)	
					A	B	A	B	A (train)	B (test)
28	*D* = −0.715(TL) −0.038(LW) +27.631	0.99	0.02	<0.001	75.0 (12)	100 (4)	90.0 (10)	75.0 (4)	92.2 (81.7–100)	87.2 (62.5–100)
42	*D* = −1.063(TL) +40.057	0.99	0.01	<0.001	100 (12)	100 (4)	100 (10)	100 (4)	99.9 (100–100)	100 (100–100)

TL: tarsus length (mm); LW: live body weight (g); A: training dataset (70 % of cases); B: test dataset (30 % of cases). The number of individuals in each sample is reported within brackets.

**Fig. 5:**
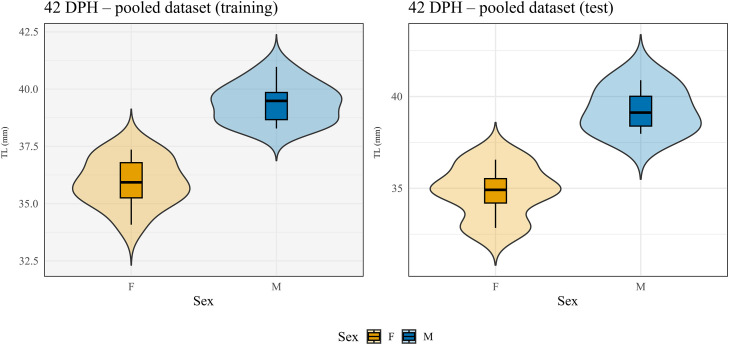
**Trait distribution by sex at 42 days post hatching (DPH) in the pooled dataset used for 70/30 training-test split. Panel A show the 70 % training subset and Panel B the 30 % test subset.** Violin plots represent the distribution of tarsus length (TL) for males and females, with violin width proportional to the relative frequency of observations. Embedded boxplots display the median (central line), interquartile range (box), and data dispersion up to 1.5 times the interquartile range (whiskers). TL was the only robust and DRRS trait retained at 42 DPH in the pooled discriminant analysis, allowing perfect sex classification in both training and test samples. F: female; M: male.

At 42 DPH, discriminant analysis retained only TL for the univariate model (Fig. 5). This function achieved perfect classification accuracy (100 %) in both training and external test samples, and bootstrap validation further supported its reliability (mean accuracy = 99.9 %, 95 % CI: 95.6–100 %), confirming the strong discriminant capacity of TL at this developmental stage. Although perfect classification accuracy was achieved at 42 DPH in this dataset, these results should be interpreted within the context of the controlled experimental design, particularly given the limited sample size, and reduced genetic variability.

## Conclusions

This study demonstrates that reliable sex determination in juvenile Rock partridges can be achieved at an early developmental stage using simple morphometric traits. Across contrasting rearing environments, tarsus length (TL) consistently emerged as the most informative and robust variable, enabling accurate sex discrimination from 42 days post-hatching. Notably, its performance was stable regardless of the rearing environment, highlighting its suitability as a rapid, cost-effective, and context-independent tool for practical management applications.

Rather than proposing a universally applicable discriminant model, this study outlines a methodological framework for identifying sex-discriminatory morphometric traits that remain robust to rearing-related environmental variation, using *Alectoris graeca* as a case study.

All individuals originated from a single wild breeding pair; a deliberate design choice aimed at minimising genetic variability and reducing captivity-related bias. While this approach strengthens inference on the effects of rearing conditions, it limits generalisation across genetic lineages. Accordingly, model performance should be interpreted as transferable across rearing environments within a controlled genetic background. Further validation involving multiple wild parental pairs or first-generation wild individuals will be required to assess the broader applicability of the proposed framework.

## Ethical statement

All procedures involving animals were conducted in compliance with relevant European and national legislation and in accordance with institutional guidelines for animal care and management. The study was based exclusively on routine husbandry, handling and management practices and did not involve any invasive procedures or experimental interventions. Consequently, ethical approval by an institutional animal care and use committee was not required.

## Data availability

All datasets generated and analysed during the current study, together with the R code used for the analyses and the additional figures, are provided in the Supplementary Material.

## Funding

This research did not receive any specific grant from funding agencies in the public, commercial, or not-for-profit sectors.

## CRediT authorship contribution statement

**Paolo Viola:** Writing – review & editing, Writing – original draft, Visualization, Supervision, Software, Methodology, Investigation, Formal analysis, Data curation, Conceptualization. **Pedro Girotti:** Writing – review & editing, Software, Data curation. **Pier Paolo Danieli:** Writing – review & editing, Validation, Data curation. **Marco Zaccaroni:** Writing – review & editing, Formal analysis. **Bruno Ronchi:** Writing – review & editing. **Nadia Piscopo:** Writing – review & editing. **Livia Lucentini:** Writing – review & editing. **Riccardo Primi:** Writing – review & editing, Writing – original draft, Validation, Supervision, Software, Resources, Methodology, Formal analysis, Conceptualization.

## Declaration of competing interest

The authors declare that they have no known competing financial interests or personal relationships that could have appeared to influence the work reported in this paper.

## Appendix Supplementary materials

Image, application 1

Image, application 2
