# Supplementary material for: Tarsus length as a simple and robust candidate for early sex determination in partridges across contrasting growing contexts: a case study in Rock partridge (Alectoris graeca Meisner, 1804)
Source: Vet Anim Sci. 2026 Apr 12;32:100657. doi: 10.1016/j.vas.2026.100657 (PMC13101291; doi:10.1016/j.vas.2026.100657)
Supplement: Supplementary file 1 [file mmc1.docx]

# Dataset

| **ID** | **Rearing system** | **Week of life** | **Days after hatching** | **Sex** | **LW** | **TL** | **TD** | **TW** | **HW** | **HL** |
| --- | --- | --- | --- | --- | --- | --- | --- | --- | --- | --- |
| 1 | G1 | D | 28 | M | 150,22 | 33,04 | 3,72 | 5,35 | 17,32 | 28,91 |
| 3 | G1 | D | 28 | M | 127,70 | 32,54 | 3,58 | 5,32 | 16,42 | 30,19 |
| 4 | G1 | D | 28 | F | 120,43 | 30,51 | 3,48 | 4,94 | 16,36 | 28,40 |
| 5 | G1 | D | 28 | F | 129,13 | 30,05 | 3,63 | 5,18 | 16,18 | 28,44 |
| 6 | G1 | D | 28 | F | 97,09 | 28,37 | 3,09 | 4,61 | 15,50 | 27,37 |
| 7 | G1 | D | 28 | M | 139,80 | 34,25 | 3,59 | 5,40 | 17,35 | 30,00 |
| 8 | G1 | D | 28 | M | 148,19 | 34,45 | 3,89 | 5,91 | 17,14 | 31,07 |
| 11 | G1 | D | 28 | M | 139,58 | 34,15 | 3,94 | 5,63 | 16,19 | 29,55 |
| 12 | G1 | D | 28 | F | 127,08 | 30,60 | 3,29 | 4,90 | 15,74 | 29,48 |
| 13 | G1 | D | 28 | F | 105,71 | 32,37 | 3,62 | 5,71 | 16,07 | 29,56 |
| 17 | G1 | D | 28 | M | 132,71 | 33,26 | 3,84 | 5,35 | 16,13 | 29,36 |
| 18 | G1 | D | 28 | F | 124,26 | 31,44 | 3,33 | 5,04 | 15,89 | 27,51 |
| 1 | G2 | D | 28 | M | 157,63 | 32,22 | 3,36 | 5,43 | 14,95 | 28,39 |
| 6 | G2 | D | 28 | M | 160,13 | 33,01 | 3,94 | 5,12 | 14,79 | 28,45 |
| 11 | G2 | D | 28 | M | 125,55 | 32,08 | 3,75 | 5,46 | 14,57 | 28,69 |
| 12 | G2 | D | 28 | M | 120,87 | 31,83 | 3,66 | 5,22 | 16,14 | 28,04 |
| 14 | G2 | D | 28 | M | 143,61 | 33,94 | 4,33 | 5,76 | 15,53 | 29,79 |
| 17 | G2 | D | 28 | F | 98,13 | 30,65 | 3,26 | 4,95 | 15,41 | 27,36 |
| 19 | G2 | D | 28 | F | 110,57 | 31,40 | 3,61 | 4,95 | 15,59 | 29,30 |
| 21 | G2 | D | 28 | F | 133,24 | 31,54 | 3,93 | 5,34 | 14,88 | 29,39 |
| 23 | G2 | D | 28 | M | 126,94 | 31,95 | 3,50 | 5,10 | 14,58 | 28,32 |
| 25 | G2 | D | 28 | F | 106,93 | 30,99 | 3,47 | 5,32 | 15,05 | 28,04 |

**Supplementary Table S1.** Individual identification code (ID), rearing system (intensive system, G1; wild-like system, G2), week of life (coded by letters from A to F, where A indicates the first week of life and F the sixth), age in days after hatching (DPH), sex, and morphometric measurements of *Alectoris graeca* juveniles at 28 days post-hatching. Live weight (LW, g), tarsus length (TL, mm), tarsus depth (TD, mm), tarsus width (TW, mm), head width (HW, mm), and head length (HL, mm) are reported for each individual. Data refer to the 70% subset of the overall dataset used to train the discriminant models.

| **ID** | **Rearing system** | **Week of life** | **Days after hatching** | **Sex** | **LW** | **TL** | **TD** | **TW** | **HW** | **HL** |
| --- | --- | --- | --- | --- | --- | --- | --- | --- | --- | --- |
| 1 | G1 | F | 42 | M | 231,59 | 40,25 | 4,13 | 5,94 | 18,59 | 32,38 |
| 3 | G1 | F | 42 | M | 233,91 | 38,72 | 4,07 | 6,26 | 18,1 | 31,82 |
| 4 | G1 | F | 42 | F | 206,48 | 35,29 | 3,61 | 5,57 | 18,01 | 30,90 |
| 5 | G1 | F | 42 | F | 201,3 | 34,08 | 3,82 | 5,71 | 16,33 | 31,53 |
| 6 | G1 | F | 42 | F | 184,91 | 35,24 | 3,77 | 5,73 | 17,27 | 30,40 |
| 7 | G1 | F | 42 | M | 223,38 | 40,33 | 4,03 | 6,3 | 19,33 | 32,10 |
| 8 | G1 | F | 42 | M | 268,83 | 40,97 | 4,53 | 6,88 | 18,48 | 32,57 |
| 11 | G1 | F | 42 | M | 256,31 | 39,71 | 4,6 | 6,71 | 19,01 | 32,86 |
| 12 | G1 | F | 42 | F | 202,54 | 35,88 | 4,06 | 5,83 | 18,79 | 30,94 |
| 13 | G1 | F | 42 | F | 224,35 | 35,17 | 3,93 | 6,21 | 18,06 | 31,66 |
| 17 | G1 | F | 42 | M | 200,27 | 38,95 | 4,06 | 6,15 | 18,33 | 33,32 |
| 18 | G1 | F | 42 | F | 214,93 | 37,36 | 3,99 | 6,22 | 18,47 | 31,99 |
| 1 | G2 | F | 42 | M | 202,01 | 39,57 | 4,37 | 6,3 | 15,82 | 32,11 |
| 6 | G2 | F | 42 | M | 197,58 | 38,51 | 4,21 | 6,12 | 16,46 | 32,23 |
| 11 | G2 | F | 42 | M | 218,2 | 38,38 | 4,17 | 6,57 | 16,49 | 32,15 |
| 12 | G2 | F | 42 | M | 213,73 | 39,4 | 4,68 | 6,79 | 17,31 | 33,50 |
| 14 | G2 | F | 42 | M | 248,43 | 39,72 | 4,64 | 6,86 | 16,37 | 32,88 |
| 17 | G2 | F | 42 | F | 179,58 | 36,21 | 3,85 | 6,1 | 15,87 | 31,00 |
| 19 | G2 | F | 42 | F | 218,34 | 35,98 | 4,32 | 5,88 | 16,34 | 32,33 |
| 21 | G2 | F | 42 | F | 224,23 | 37,17 | 4,11 | 6,18 | 16,11 | 32,04 |
| 23 | G2 | F | 42 | M | 218,97 | 38,28 | 4,08 | 6,33 | 15,89 | 32,13 |
| 25 | G2 | F | 42 | F | 196,58 | 36,98 | 4,04 | 6,1 | 15,47 | 31,56 |

**Supplementary Table S2.** Individual identification code (ID), rearing system (intensive system, G1; wild-like system, G2), week of life (coded by letters from A to F, where A indicates the first week of life and F the sixth), age in days after hatching (DPH), sex, and morphometric measurements of *Alectoris graeca* juveniles at 42 days post-hatching. Live weight (LW, g), tarsus length (TL, mm), tarsus depth (TD, mm), tarsus width (TW, mm), head width (HW, mm), and head length (HL, mm) are reported for each individual. Data refer to the 70% subset of the overall dataset used to train the discriminant models.

| **ID** | **Rearing system** | **Week of life** | **Days after hatching** | **Sex** | **LW** | **TL** | **TD** | **TW** | **HW** | **HL** |
| --- | --- | --- | --- | --- | --- | --- | --- | --- | --- | --- |
| 10 | G1 | D | 28 | F | 124,29 | 30,68 | 3,47 | 5,32 | 15,19 | 29,41 |
| 16 | G1 | D | 28 | F | 118,56 | 32,61 | 3,24 | 5,3 | 15,64 | 29,33 |
| 15 | G1 | D | 28 | M | 147,8 | 34,29 | 3,81 | 5,45 | 15,88 | 30,07 |
| 2 | G1 | D | 28 | M | 149,27 | 33,58 | 3,59 | 5,48 | 16,11 | 29,78 |
| 15 | G2 | D | 28 | F | 83,4 | 30,33 | 3,32 | 4,6 | 15,64 | 27,04 |
| 9 | G2 | D | 28 | F | 112,28 | 30,86 | 3,53 | 5,17 | 14,44 | 29,51 |
| 13 | G2 | D | 28 | M | 152,87 | 32,98 | 3,45 | 5,1 | 15,49 | 31,63 |
| 22 | G2 | D | 28 | M | 127,86 | 32,99 | 3,6 | 5,38 | 15,89 | 29,23 |

**Supplementary Table S3.** Individual identification code (ID), rearing system (intensive system, G1; wild-like system, G2), week of life (coded by letters from A to F, where A indicates the first week of life and F the sixth), age in days after hatching (DPH), sex, and morphometric measurements of *Alectoris graeca* juveniles at 28 days post-hatching. Live weight (LW, g), tarsus length (TL, mm), tarsus depth (TD, mm), tarsus width (TW, mm), head width (HW, mm), and head length (HL, mm) are reported for each individual. Data refer to the 30% subset of the overall dataset used for external validation of the discriminant models.

| **ID** | **Rearing system** | **Week of life** | **Days after hatching** | **Sex** | **LW** | **TL** | **TD** | **TW** | **HW** | **HL** |
| --- | --- | --- | --- | --- | --- | --- | --- | --- | --- | --- |
| 10 | G1 | F | 42 | F | 201,21 | 32,84 | 4,06 | 6,24 | 18,16 | 31,64 |
| 16 | G1 | F | 42 | F | 218,92 | 35,18 | 3,71 | 6,04 | 18,07 | 31,80 |
| 15 | G1 | F | 42 | M | 250,23 | 40,89 | 4,24 | 6,56 | 18,8 | 32,67 |
| 2 | G1 | F | 42 | M | 236,04 | 37,97 | 3,93 | 6,4 | 18,32 | 32,76 |
| 15 | G2 | F | 42 | F | 139 | 34,65 | 3,62 | 5,03 | 16,05 | 30,58 |
| 9 | G2 | F | 42 | F | 209,98 | 36,56 | 4,35 | 6,09 | 15,89 | 30,37 |
| 13 | G2 | F | 42 | M | 198,2 | 38,53 | 4,37 | 6,37 | 16,24 | 32,53 |
| 22 | G2 | F | 42 | M | 227,5 | 39,72 | 4,34 | 6,47 | 17,12 | 32,52 |

**Supplementary Table S4.** Individual identification code (ID), rearing system (intensive system, G1; wild-like system, G2), week of life (coded by letters from A to F, where A indicates the first week of life and F the sixth), age in days after hatching (DPH), sex, and morphometric measurements of *Alectoris graeca* juveniles at 42 days post-hatching. Live weight (LW, g), tarsus length (TL, mm), tarsus depth (TD, mm), tarsus width (TW, mm), head width (HW, mm), and head length (HL, mm) are reported for each individual. Data refer to the 30% subset of the overall dataset used for external validation of the discriminant models.

# LDA MODEL (PAPER-READY) - 28 DPH (stepwise – full dataset)

## STEPWISE LDA OUTPUT (F-enter / F-remove)

=============================================

STEPWISE LDA (SPSS-like: F-enter/F-remove)

=============================================

Initial candidate traits:

[1] "TL" "LW" "TD" "HL" "TW"

F to enter = 4 F to remove = 3.9 Tolerance = 0.001

>>> FORWARD STEP

F_enter and statistics for all candidate variables:

Var Wilks(full) Wilks(minus) Partial Wilks F df1 df2 Tol(min)

TL 0.0339 1.0000 0.0339 398.704 1 14 NA

LW 0.0422 1.0000 0.0422 317.865 1 14 NA

TD 0.0546 1.0000 0.0546 242.310 1 14 NA

HL 0.0983 1.0000 0.0983 128.432 1 14 NA

TW 0.1251 1.0000 0.1251 97.909 1 14 NA

Best candidate to enter: TL

Partial F (enter) = 398.704

✔ Variable ENTERED: TL

✔ Currently selected variables:

[1] "TL"

>>> FORWARD STEP

F_enter and statistics for all candidate variables:

Var Wilks(full) Wilks(minus) Partial Wilks F df1 df2 Tol(min)

LW 0.0247 0.0339 0.7293 4.824 1 13 0.4359

TD 0.0284 0.0339 0.8373 2.525 1 13 0.4412

HL 0.0339 0.0339 0.9996 0.005 1 13 0.4210

TW 0.0292 0.0339 0.8618 2.084 1 13 0.3128

Best candidate to enter: LW

Partial F (enter) = 4.824

✔ Variable ENTERED: LW

✔ Currently selected variables:

[1] "TL" "LW"

>>> BACKWARD STEP

F_remove and statistics for all variables in the model:

Var Wilks(full) Wilks(minus) Partial Wilks F df1 df2 Tol(min)

TL 0.0247 0.0422 0.5865 9.166 1 13 NA

LW 0.0247 0.0339 0.7293 4.824 1 13 NA

Least significant variable in the model: LW

Partial F (remove) = 4.824

No variable meets the removal criterion.

>>> FORWARD STEP

F_enter and statistics for all candidate variables:

Var Wilks(full) Wilks(minus) Partial Wilks F df1 df2 Tol(min)

TD 0.0230 0.0247 0.9286 0.922 1 12 0.3409

HL 0.0247 0.0247 0.9999 0.002 1 12 0.2757

TW 0.0226 0.0247 0.9124 1.152 1 12 0.1880

Best candidate to enter: TW

Partial F (enter) = 1.152

No variable meets the F_enter criterion.

>>> BACKWARD STEP

F_remove and statistics for all variables in the model:

Var Wilks(full) Wilks(minus) Partial Wilks F df1 df2 Tol(min)

TL 0.0247 0.0422 0.5865 9.166 1 13 NA

LW 0.0247 0.0339 0.7293 4.824 1 13 NA

Least significant variable in the model: LW

Partial F (remove) = 4.824

No variable meets the removal criterion.

>>> No further changes: stepwise procedure completed.

Final selected variables:

[1] "TL" "LW"

## LDA PIPELINE OUTPUT

=============================================

LDA PIPELINE - 28 DPH (stepwise – full dataset)

=============================================

→ Classes (Sex): F M

→ Equal priors: 0.5 0.5

→ male_lev = M , female_lev = F

▶ Box's M test for homogeneity of covariance matrices:

Box's M-test for Homogeneity of Covariance Matrices

data: lda_data_G1[, traits, drop = FALSE]

Chi-Sq (approx.) = 3.732, df = 3, p-value = 0.2919

(conservative significance threshold p = 0.01)

▶ LDA model (training sample - 28 DPH (stepwise – full dataset) )

Call:

lda(form, data = lda_data_G1, prior = priors_equal)

Prior probabilities of groups:

F M

0.5 0.5

Group means:

TL LW

F 30.82875 118.3187

M 33.69500 141.9087

Coefficients of linear discriminants:

LD1

TL 0.63588551

LW 0.05581104

▶ Canonical correlations (r):

[1] 0.9875518

▶ Global Wilks' Lambda: 0.0247

▶ Stratified bootstrap (1000 iterations) on training sample - 28 DPH (stepwise – full dataset)

- Mean accuracy (bootstrap, training sample, overall): 97.91 %

- Mean accuracy (bootstrap, training sample, M): 96.94 %

- Mean accuracy (bootstrap, training sample, F): 98.88 %

- 95% CI (bootstrap, training sample, overall): 87.5 % - 100 %

- Mean #correct M (bootstrap, training): 7.76 su 8 [95% CI: 6 – 8 ]

- Mean #correct F (bootstrap, training): 7.91 su 8 [95% CI: 7 – 8 ]

▶ Jackknife cross-validation (LOOCV) on training sample - 28 DPH (stepwise – full dataset)

- Jackknife accuracy (training sample): 93.75 %

- Confusion matrix (Jackknife, training sample):

Actual

Predicted F M

F 8 1

M 0 7

▶ Final test on external test sample - 28 DPH (stepwise – full dataset)

- Test accuracy (external test sample, overall): 78.57 %

- Confusion matrix (external test sample):

Actual

Predicted F M

F 6 3

M 0 5

▶ Stratified bootstrap (1000 iterations) on external test sample - 28 DPH (stepwise – full dataset)

- Mean accuracy (bootstrap, external test sample, overall): 78.72 %

- Mean accuracy (bootstrap, external test sample, M): 62.76 %

- Mean accuracy (bootstrap, external test sample, F): 100 %

- 95% CI (bootstrap, external test sample, overall): 57.14 % - 92.86 %

- Mean #correct M (bootstrap, external test): 5.02 su 8 [95% CI: 2 – 7 ]

- Mean #correct F (bootstrap, external test): 6 su 6 [95% CI: 6 – 6 ]

## LDA SUMMARY (PAPER-READY, D > 0 = female)

| Parameter | Value |
| --- | --- |
| Discriminant function (paper-ready) | D = -0.636(TL) -0.056(LW) +27.777 |
| LD1 raw coefficients | TL 0.636; LW 0.056 |
| Coefficients used in D (paper-ready) | TL -0.636; LW -0.056 |
| Intercept | 27.777 |
| Canonical r (MASS raw / SVD) | 0.99 |
| Canonical r (from Wilks raw) | 0.99 |
| Canonical r (SPSS-like, reported) | 0.86 |
| Wilks' lambda (MASS raw) | 0.025 |
| Wilks' lambda (SPSS-like, reported) | 0.262 |
| p-value (MASS raw) | <0.001 |
| p-value (SPSS-like, reported) | <0.001 |
| Train M (%) | 87.5 (n=8) |
| Train F (%) | 100 (n=8) |
| Test M (%) | 62.5 (n=8) |
| Test F (%) | 100 (n=6) |
| Bootstrap train acc (mean, 95% CI) | 97.91% (87.50–100.00%) |
| Bootstrap test acc (mean, 95% CI) | 78.72% (57.14–92.86%) |

### Correct classification (G1 – TRAIN / Jackknife)

- Males: 87.5 % (n = 8)

- Females: 100.0 % (n = 8)

### Correct classification (G2 – TEST)

- Males: 62.5 % (n = 8)

- Females: 100.0 % (n = 6)

# LDA MODEL (PAPER-READY) - 42 DPH (stepwise – full dataset)

## STEPWISE LDA OUTPUT (F-enter / F-remove)

=============================================

STEPWISE LDA (SPSS-like: F-enter/F-remove)

=============================================

Initial candidate traits:

[1] "TL" "LW" "TD" "HL" "TW"

F to enter = 4 F to remove = 3.9 Tolerance = 0.001

>>> FORWARD STEP

F_enter and statistics for all candidate variables:

Var Wilks(full) Wilks(minus) Partial Wilks F df1 df2 Tol(min)

TL 0.0168 1.0000 0.0168 820.508 1 14 NA

LW 0.0737 1.0000 0.0737 175.858 1 14 NA

TD 0.0909 1.0000 0.0909 140.061 1 14 NA

HL 0.0427 1.0000 0.0427 314.144 1 14 NA

TW 0.0900 1.0000 0.0900 141.546 1 14 NA

Best candidate to enter: TL

Partial F (enter) = 820.508

✔ Variable ENTERED: TL

✔ Currently selected variables:

[1] "TL"

>>> FORWARD STEP

F_enter and statistics for all candidate variables:

Var Wilks(full) Wilks(minus) Partial Wilks F df1 df2 Tol(min)

LW 0.0166 0.0168 0.9912 0.115 1 13 0.4563

TD 0.0165 0.0168 0.9824 0.233 1 13 0.5442

HL 0.0118 0.0168 0.7031 5.489 1 13 0.5167

TW 0.0158 0.0168 0.9445 0.764 1 13 0.5901

Best candidate to enter: HL

Partial F (enter) = 5.489

✔ Variable ENTERED: HL

✔ Currently selected variables:

[1] "TL" "HL"

>>> BACKWARD STEP

F_remove and statistics for all variables in the model:

Var Wilks(full) Wilks(minus) Partial Wilks F df1 df2 Tol(min)

TL 0.0118 0.0427 0.2765 34.019 1 13 NA

HL 0.0118 0.0168 0.7031 5.489 1 13 NA

Least significant variable in the model: HL

Partial F (remove) = 5.489

No variable meets the removal criterion.

>>> FORWARD STEP

F_enter and statistics for all candidate variables:

Var Wilks(full) Wilks(minus) Partial Wilks F df1 df2 Tol(min)

LW 0.0118 0.0118 0.9975 0.031 1 12 0.3686

TD 0.0118 0.0118 0.9990 0.012 1 12 0.4202

TW 0.0118 0.0118 0.9968 0.039 1 12 0.3870

Best candidate to enter: TW

Partial F (enter) = 0.039

No variable meets the F_enter criterion.

>>> BACKWARD STEP

F_remove and statistics for all variables in the model:

Var Wilks(full) Wilks(minus) Partial Wilks F df1 df2 Tol(min)

TL 0.0118 0.0427 0.2765 34.019 1 13 NA

HL 0.0118 0.0168 0.7031 5.489 1 13 NA

Least significant variable in the model: HL

Partial F (remove) = 5.489

No variable meets the removal criterion.

>>> No further changes: stepwise procedure completed.

Final selected variables:

[1] "TL" "HL"

## LDA PIPELINE OUTPUT

=============================================

LDA PIPELINE - 42 DPH (stepwise – full dataset)

=============================================

→ Classes (Sex): F M

→ Equal priors: 0.5 0.5

→ male_lev = M , female_lev = F

▶ Box's M test for homogeneity of covariance matrices:

Box's M-test for Homogeneity of Covariance Matrices

data: lda_data_G1[, traits, drop = FALSE]

Chi-Sq (approx.) = 0.43697, df = 3, p-value = 0.9325

(conservative significance threshold p = 0.01)

▶ LDA model (training sample - 42 DPH (stepwise – full dataset) )

Call:

lda(form, data = lda_data_G1, prior = priors_equal)

Prior probabilities of groups:

F M

0.5 0.5

Group means:

TL HL

F 35.13000 31.3575

M 39.72375 32.5600

Coefficients of linear discriminants:

LD1

TL 0.7134506

HL 1.0802576

▶ Canonical correlations (r):

[1] 0.9940845

▶ Global Wilks' Lambda: 0.0118

▶ Stratified bootstrap (1000 iterations) on training sample - 42 DPH (stepwise – full dataset)

- Mean accuracy (bootstrap, training sample, overall): 98.74 %

- Mean accuracy (bootstrap, training sample, M): 99.95 %

- Mean accuracy (bootstrap, training sample, F): 97.54 %

- 95% CI (bootstrap, training sample, overall): 93.75 % - 100 %

- Mean #correct M (bootstrap, training): 8 su 8 [95% CI: 8 – 8 ]

- Mean #correct F (bootstrap, training): 7.8 su 8 [95% CI: 7 – 8 ]

▶ Jackknife cross-validation (LOOCV) on training sample - 42 DPH (stepwise – full dataset)

- Jackknife accuracy (training sample): 93.75 %

- Confusion matrix (Jackknife, training sample):

Actual

Predicted F M

F 7 0

M 1 8

▶ Final test on external test sample - 42 DPH (stepwise – full dataset)

- Test accuracy (external test sample, overall): 100 %

- Confusion matrix (external test sample):

Actual

Predicted F M

F 6 0

M 0 8

▶ Stratified bootstrap (1000 iterations) on external test sample - 42 DPH (stepwise – full dataset)

- Mean accuracy (bootstrap, external test sample, overall): 100 %

- Mean accuracy (bootstrap, external test sample, M): 100 %

- Mean accuracy (bootstrap, external test sample, F): 100 %

- 95% CI (bootstrap, external test sample, overall): 100 % - 100 %

- Mean #correct M (bootstrap, external test): 8 su 8 [95% CI: 8 – 8 ]

- Mean #correct F (bootstrap, external test): 6 su 6 [95% CI: 6 – 6 ]

## LDA SUMMARY (PAPER-READY, D > 0 = female)

| Parameter | Value |
| --- | --- |
| Discriminant function (paper-ready) | D = -0.713(TL) -1.080(HL) +61.226 |
| LD1 raw coefficients | TL 0.713; HL 1.08 |
| Coefficients used in D (paper-ready) | TL -0.713; HL -1.08 |
| Intercept | 61.226 |
| Canonical r (MASS raw / SVD) | 0.99 |
| Canonical r (from Wilks raw) | 0.99 |
| Canonical r (SPSS-like, reported) | 0.93 |
| Wilks' lambda (MASS raw) | 0.012 |
| Wilks' lambda (SPSS-like, reported) | 0.143 |
| p-value (MASS raw) | <0.001 |
| p-value (SPSS-like, reported) | <0.001 |
| Train M (%) | 100 (n=8) |
| Train F (%) | 87.5 (n=8) |
| Test M (%) | 100 (n=8) |
| Test F (%) | 100 (n=6) |
| Bootstrap train acc (mean, 95% CI) | 98.74% (93.75–100.00%) |
| Bootstrap test acc (mean, 95% CI) | 100.00% (100.00–100.00%) |

### Correct classification (G1 – TRAIN / Jackknife)

- Males: 100.0 % (n = 8)

- Females: 87.5 % (n = 8)

### Correct classification (G2 – TEST)

- Males: 100.0 % (n = 8)

- Females: 100.0 % (n = 6)

# LDA MODEL (PAPER-READY) - 28 DPH (stepwise – TL, LW)

## STEPWISE LDA OUTPUT (F-enter / F-remove)

=============================================

STEPWISE LDA (SPSS-like: F-enter/F-remove)

=============================================

Initial candidate traits:

[1] "TL" "LW"

F to enter = 4 F to remove = 3.9 Tolerance = 0.001

>>> FORWARD STEP

F_enter and statistics for all candidate variables:

Var Wilks(full) Wilks(minus) Partial Wilks F df1 df2 Tol(min)

TL 0.0339 1.0000 0.0339 398.704 1 14 NA

LW 0.0422 1.0000 0.0422 317.865 1 14 NA

Best candidate to enter: TL

Partial F (enter) = 398.704

✔ Variable ENTERED: TL

✔ Currently selected variables:

[1] "TL"

>>> FORWARD STEP

F_enter and statistics for all candidate variables:

Var Wilks(full) Wilks(minus) Partial Wilks F df1 df2 Tol(min)

LW 0.0247 0.0339 0.7293 4.824 1 13 0.4359

Best candidate to enter: LW

Partial F (enter) = 4.824

✔ Variable ENTERED: LW

✔ Currently selected variables:

[1] "TL" "LW"

>>> BACKWARD STEP

F_remove and statistics for all variables in the model:

Var Wilks(full) Wilks(minus) Partial Wilks F df1 df2 Tol(min)

TL 0.0247 0.0422 0.5865 9.166 1 13 NA

LW 0.0247 0.0339 0.7293 4.824 1 13 NA

Least significant variable in the model: LW

Partial F (remove) = 4.824

No variable meets the removal criterion.

>>> BACKWARD STEP

F_remove and statistics for all variables in the model:

Var Wilks(full) Wilks(minus) Partial Wilks F df1 df2 Tol(min)

TL 0.0247 0.0422 0.5865 9.166 1 13 NA

LW 0.0247 0.0339 0.7293 4.824 1 13 NA

Least significant variable in the model: LW

Partial F (remove) = 4.824

No variable meets the removal criterion.

>>> No further changes: stepwise procedure completed.

Final selected variables:

[1] "TL" "LW"

## LDA PIPELINE OUTPUT

=============================================

LDA PIPELINE - 28 DPH (stepwise – TL, LW)

=============================================

→ Classes (Sex): F M

→ Equal priors: 0.5 0.5

→ male_lev = M , female_lev = F

▶ Box's M test for homogeneity of covariance matrices:

Box's M-test for Homogeneity of Covariance Matrices

data: lda_data_G1[, traits, drop = FALSE]

Chi-Sq (approx.) = 3.732, df = 3, p-value = 0.2919

(conservative significance threshold p = 0.01)

▶ LDA model (training sample - 28 DPH (stepwise – TL, LW) )

Call:

lda(form, data = lda_data_G1, prior = priors_equal)

Prior probabilities of groups:

F M

0.5 0.5

Group means:

TL LW

F 30.82875 118.3187

M 33.69500 141.9087

Coefficients of linear discriminants:

LD1

TL 0.63588551

LW 0.05581104

▶ Canonical correlations (r):

[1] 0.9875518

▶ Global Wilks' Lambda: 0.0247

▶ Stratified bootstrap (1000 iterations) on training sample - 28 DPH (stepwise – TL, LW)

- Mean accuracy (bootstrap, training sample, overall): 97.91 %

- Mean accuracy (bootstrap, training sample, M): 96.94 %

- Mean accuracy (bootstrap, training sample, F): 98.88 %

- 95% CI (bootstrap, training sample, overall): 87.5 % - 100 %

- Mean #correct M (bootstrap, training): 7.76 su 8 [95% CI: 6 – 8 ]

- Mean #correct F (bootstrap, training): 7.91 su 8 [95% CI: 7 – 8 ]

▶ Jackknife cross-validation (LOOCV) on training sample - 28 DPH (stepwise – TL, LW)

- Jackknife accuracy (training sample): 93.75 %

- Confusion matrix (Jackknife, training sample):

Actual

Predicted F M

F 8 1

M 0 7

▶ Final test on external test sample - 28 DPH (stepwise – TL, LW)

- Test accuracy (external test sample, overall): 78.57 %

- Confusion matrix (external test sample):

Actual

Predicted F M

F 6 3

M 0 5

▶ Stratified bootstrap (1000 iterations) on external test sample - 28 DPH (stepwise – TL, LW)

- Mean accuracy (bootstrap, external test sample, overall): 78.72 %

- Mean accuracy (bootstrap, external test sample, M): 62.76 %

- Mean accuracy (bootstrap, external test sample, F): 100 %

- 95% CI (bootstrap, external test sample, overall): 57.14 % - 92.86 %

- Mean #correct M (bootstrap, external test): 5.02 su 8 [95% CI: 2 – 7 ]

- Mean #correct F (bootstrap, external test): 6 su 6 [95% CI: 6 – 6 ]

## LDA SUMMARY (PAPER-READY, D > 0 = female)

| Parameter | Value |
| --- | --- |
| Discriminant function (paper-ready) | D = -0.636(TL) -0.056(LW) +27.777 |
| LD1 raw coefficients | TL 0.636; LW 0.056 |
| Coefficients used in D (paper-ready) | TL -0.636; LW -0.056 |
| Intercept | 27.777 |
| Canonical r (MASS raw / SVD) | 0.99 |
| Canonical r (from Wilks raw) | 0.99 |
| Canonical r (SPSS-like, reported) | 0.86 |
| Wilks' lambda (MASS raw) | 0.025 |
| Wilks' lambda (SPSS-like, reported) | 0.262 |
| p-value (MASS raw) | <0.001 |
| p-value (SPSS-like, reported) | <0.001 |
| Train M (%) | 87.5 (n=8) |
| Train F (%) | 100 (n=8) |
| Test M (%) | 62.5 (n=8) |
| Test F (%) | 100 (n=6) |
| Bootstrap train acc (mean, 95% CI) | 97.91% (87.50–100.00%) |
| Bootstrap test acc (mean, 95% CI) | 78.72% (57.14–92.86%) |

### Correct classification (G1 – TRAIN / Jackknife)

- Males: 87.5 % (n = 8)

- Females: 100.0 % (n = 8)

### Correct classification (G2 – TEST)

- Males: 62.5 % (n = 8)

- Females: 100.0 % (n = 6)

# LDA MODEL (PAPER-READY) - 42 DPH (stepwise – TL, HL)

## STEPWISE LDA OUTPUT (F-enter / F-remove)

=============================================

STEPWISE LDA (SPSS-like: F-enter/F-remove)

=============================================

Initial candidate traits:

[1] "TL" "HL"

F to enter = 4 F to remove = 3.9 Tolerance = 0.001

>>> FORWARD STEP

F_enter and statistics for all candidate variables:

Var Wilks(full) Wilks(minus) Partial Wilks F df1 df2 Tol(min)

TL 0.0168 1.0000 0.0168 820.508 1 14 NA

HL 0.0427 1.0000 0.0427 314.144 1 14 NA

Best candidate to enter: TL

Partial F (enter) = 820.508

✔ Variable ENTERED: TL

✔ Currently selected variables:

[1] "TL"

>>> FORWARD STEP

F_enter and statistics for all candidate variables:

Var Wilks(full) Wilks(minus) Partial Wilks F df1 df2 Tol(min)

HL 0.0118 0.0168 0.7031 5.489 1 13 0.5167

Best candidate to enter: HL

Partial F (enter) = 5.489

✔ Variable ENTERED: HL

✔ Currently selected variables:

[1] "TL" "HL"

>>> BACKWARD STEP

F_remove and statistics for all variables in the model:

Var Wilks(full) Wilks(minus) Partial Wilks F df1 df2 Tol(min)

TL 0.0118 0.0427 0.2765 34.019 1 13 NA

HL 0.0118 0.0168 0.7031 5.489 1 13 NA

Least significant variable in the model: HL

Partial F (remove) = 5.489

No variable meets the removal criterion.

>>> BACKWARD STEP

F_remove and statistics for all variables in the model:

Var Wilks(full) Wilks(minus) Partial Wilks F df1 df2 Tol(min)

TL 0.0118 0.0427 0.2765 34.019 1 13 NA

HL 0.0118 0.0168 0.7031 5.489 1 13 NA

Least significant variable in the model: HL

Partial F (remove) = 5.489

No variable meets the removal criterion.

>>> No further changes: stepwise procedure completed.

Final selected variables:

[1] "TL" "HL"

## LDA PIPELINE OUTPUT

=============================================

LDA PIPELINE - 42 DPH (stepwise – TL, HL)

=============================================

→ Classes (Sex): F M

→ Equal priors: 0.5 0.5

→ male_lev = M , female_lev = F

▶ Box's M test for homogeneity of covariance matrices:

Box's M-test for Homogeneity of Covariance Matrices

data: lda_data_G1[, traits, drop = FALSE]

Chi-Sq (approx.) = 0.43697, df = 3, p-value = 0.9325

(conservative significance threshold p = 0.01)

▶ LDA model (training sample - 42 DPH (stepwise – TL, HL) )

Call:

lda(form, data = lda_data_G1, prior = priors_equal)

Prior probabilities of groups:

F M

0.5 0.5

Group means:

TL HL

F 35.13000 31.3575

M 39.72375 32.5600

Coefficients of linear discriminants:

LD1

TL 0.7134506

HL 1.0802576

▶ Canonical correlations (r):

[1] 0.9940845

▶ Global Wilks' Lambda: 0.0118

▶ Stratified bootstrap (1000 iterations) on training sample - 42 DPH (stepwise – TL, HL)

- Mean accuracy (bootstrap, training sample, overall): 98.74 %

- Mean accuracy (bootstrap, training sample, M): 99.95 %

- Mean accuracy (bootstrap, training sample, F): 97.54 %

- 95% CI (bootstrap, training sample, overall): 93.75 % - 100 %

- Mean #correct M (bootstrap, training): 8 su 8 [95% CI: 8 – 8 ]

- Mean #correct F (bootstrap, training): 7.8 su 8 [95% CI: 7 – 8 ]

▶ Jackknife cross-validation (LOOCV) on training sample - 42 DPH (stepwise – TL, HL)

- Jackknife accuracy (training sample): 93.75 %

- Confusion matrix (Jackknife, training sample):

Actual

Predicted F M

F 7 0

M 1 8

▶ Final test on external test sample - 42 DPH (stepwise – TL, HL)

- Test accuracy (external test sample, overall): 100 %

- Confusion matrix (external test sample):

Actual

Predicted F M

F 6 0

M 0 8

▶ Stratified bootstrap (1000 iterations) on external test sample - 42 DPH (stepwise – TL, HL)

- Mean accuracy (bootstrap, external test sample, overall): 100 %

- Mean accuracy (bootstrap, external test sample, M): 100 %

- Mean accuracy (bootstrap, external test sample, F): 100 %

- 95% CI (bootstrap, external test sample, overall): 100 % - 100 %

- Mean #correct M (bootstrap, external test): 8 su 8 [95% CI: 8 – 8 ]

- Mean #correct F (bootstrap, external test): 6 su 6 [95% CI: 6 – 6 ]

## LDA SUMMARY (PAPER-READY, D > 0 = female)

| Parameter | Value |
| --- | --- |
| Discriminant function (paper-ready) | D = -0.713(TL) -1.080(HL) +61.226 |
| LD1 raw coefficients | TL 0.713; HL 1.08 |
| Coefficients used in D (paper-ready) | TL -0.713; HL -1.08 |
| Intercept | 61.226 |
| Canonical r (MASS raw / SVD) | 0.99 |
| Canonical r (from Wilks raw) | 0.99 |
| Canonical r (SPSS-like, reported) | 0.93 |
| Wilks' lambda (MASS raw) | 0.012 |
| Wilks' lambda (SPSS-like, reported) | 0.143 |
| p-value (MASS raw) | <0.001 |
| p-value (SPSS-like, reported) | <0.001 |
| Train M (%) | 100 (n=8) |
| Train F (%) | 87.5 (n=8) |
| Test M (%) | 100 (n=8) |
| Test F (%) | 100 (n=6) |
| Bootstrap train acc (mean, 95% CI) | 98.74% (93.75–100.00%) |
| Bootstrap test acc (mean, 95% CI) | 100.00% (100.00–100.00%) |

### Correct classification (G1 – TRAIN / Jackknife)

- Males: 100.0 % (n = 8)

- Females: 87.5 % (n = 8)

### Correct classification (G2 – TEST)

- Males: 100.0 % (n = 8)

- Females: 100.0 % (n = 6)

# LDA MODEL (PAPER-READY) - 28 DPH 70/30 split (stepwise)

## STEPWISE LDA OUTPUT (F-enter / F-remove)

=============================================

STEPWISE LDA (SPSS-like: F-enter/F-remove)

=============================================

Initial candidate traits:

[1] "TL" "LW"

F to enter = 4 F to remove = 3.9 Tolerance = 0.001

>>> FORWARD STEP

F_enter and statistics for all candidate variables:

Var Wilks(full) Wilks(minus) Partial Wilks F df1 df2 Tol(min)

TL 0.0349 1.0000 0.0349 552.922 1 20 NA

LW 0.0505 1.0000 0.0505 375.767 1 20 NA

Best candidate to enter: TL

Partial F (enter) = 552.922

✔ Variable ENTERED: TL

✔ Currently selected variables:

[1] "TL"

>>> FORWARD STEP

F_enter and statistics for all candidate variables:

Var Wilks(full) Wilks(minus) Partial Wilks F df1 df2 Tol(min)

LW 0.0276 0.0349 0.7904 5.039 1 19 0.5348

Best candidate to enter: LW

Partial F (enter) = 5.039

✔ Variable ENTERED: LW

✔ Currently selected variables:

[1] "TL" "LW"

>>> BACKWARD STEP

F_remove and statistics for all variables in the model:

Var Wilks(full) Wilks(minus) Partial Wilks F df1 df2 Tol(min)

TL 0.0276 0.0505 0.5460 15.799 1 19 NA

LW 0.0276 0.0349 0.7904 5.039 1 19 NA

Least significant variable in the model: LW

Partial F (remove) = 5.039

No variable meets the removal criterion.

>>> BACKWARD STEP

F_remove and statistics for all variables in the model:

Var Wilks(full) Wilks(minus) Partial Wilks F df1 df2 Tol(min)

TL 0.0276 0.0505 0.5460 15.799 1 19 NA

LW 0.0276 0.0349 0.7904 5.039 1 19 NA

Least significant variable in the model: LW

Partial F (remove) = 5.039

No variable meets the removal criterion.

>>> No further changes: stepwise procedure completed.

Final selected variables:

[1] "TL" "LW"

## LDA PIPELINE OUTPUT

=============================================

LDA PIPELINE - 28 DPH 70/30 split (stepwise)

=============================================

→ Classes (Sex): F M

→ Equal priors: 0.5 0.5

→ male_lev = M , female_lev = F

▶ Box's M test for homogeneity of covariance matrices:

Box's M-test for Homogeneity of Covariance Matrices

data: lda_data_G1[, traits, drop = FALSE]

Chi-Sq (approx.) = 0.35017, df = 3, p-value = 0.9503

(conservative significance threshold p = 0.01)

▶ LDA model (training sample - 28 DPH 70/30 split (stepwise) )

Call:

lda(form, data = lda_data_G1, prior = priors_equal)

Prior probabilities of groups:

F M

0.5 0.5

Group means:

TL LW

F 30.792 115.2570

M 33.060 139.4108

Coefficients of linear discriminants:

LD1

TL 0.71539650

LW 0.03762761

▶ Canonical correlations (r):

[1] 0.9861078

▶ Global Wilks' Lambda: 0.0276

▶ Stratified bootstrap (1000 iterations) on training sample - 28 DPH 70/30 split (stepwise)

- Mean accuracy (bootstrap, training sample, overall): 92.15 %

- Mean accuracy (bootstrap, training sample, M): 87.92 %

- Mean accuracy (bootstrap, training sample, F): 97.23 %

- 95% CI (bootstrap, training sample, overall): 81.7 % - 100 %

- Mean #correct M (bootstrap, training): 10.55 su 12 [95% CI: 8 – 12 ]

- Mean #correct F (bootstrap, training): 9.72 su 10 [95% CI: 8 – 10 ]

▶ Jackknife cross-validation (LOOCV) on training sample - 28 DPH 70/30 split (stepwise)

- Jackknife accuracy (training sample): 81.82 %

- Confusion matrix (Jackknife, training sample):

Actual

Predicted F M

F 9 3

M 1 9

▶ Final test on external test sample - 28 DPH 70/30 split (stepwise)

- Test accuracy (external test sample, overall): 87.5 %

- Confusion matrix (external test sample):

Actual

Predicted F M

F 3 0

M 1 4

▶ Stratified bootstrap (1000 iterations) on external test sample - 28 DPH 70/30 split (stepwise)

- Mean accuracy (bootstrap, external test sample, overall): 87.22 %

- Mean accuracy (bootstrap, external test sample, M): 100 %

- Mean accuracy (bootstrap, external test sample, F): 74.45 %

- 95% CI (bootstrap, external test sample, overall): 62.5 % - 100 %

- Mean #correct M (bootstrap, external test): 4 su 4 [95% CI: 4 – 4 ]

- Mean #correct F (bootstrap, external test): 2.98 su 4 [95% CI: 1 – 4 ]

## LDA SUMMARY (PAPER-READY, D > 0 = female)

| Parameter | Value |
| --- | --- |
| Discriminant function (paper-ready) | D = -0.715(TL) -0.038(LW) +27.631 |
| LD1 raw coefficients | TL 0.715; LW 0.038 |
| Coefficients used in D (paper-ready) | TL -0.715; LW -0.038 |
| Intercept | 27.631 |
| Canonical r (MASS raw / SVD) | 0.99 |
| Canonical r (from Wilks raw) | 0.99 |
| Canonical r (SPSS-like, reported) | 0.80 |
| Wilks' lambda (MASS raw) | 0.028 |
| Wilks' lambda (SPSS-like, reported) | 0.364 |
| p-value (MASS raw) | <0.001 |
| p-value (SPSS-like, reported) | <0.001 |
| Train M (%) | 75 (n=12) |
| Train F (%) | 90 (n=10) |
| Test M (%) | 100 (n=4) |
| Test F (%) | 75 (n=4) |
| Bootstrap train acc (mean, 95% CI) | 92.15% (81.70–100.00%) |
| Bootstrap test acc (mean, 95% CI) | 87.22% (62.50–100.00%) |

### Correct classification (training sample – Jackknife)

- Males: 75.0 % (n = 12)

- Females: 90.0 % (n = 10)

### Correct classification (external test sample)

- Males: 100.0 % (n = 4)

- Females: 75.0 % (n = 4)

# LDA MODEL (PAPER-READY) - 42 DPH 70/30 split (stepwise)

## STEPWISE LDA OUTPUT (F-enter / F-remove)

=============================================

STEPWISE LDA (SPSS-like: F-enter/F-remove)

=============================================

Initial candidate traits:

[1] "TL" "HL"

F to enter = 4 F to remove = 3.9 Tolerance = 0.001

>>> FORWARD STEP

F_enter and statistics for all candidate variables:

Var Wilks(full) Wilks(minus) Partial Wilks F df1 df2 Tol(min)

TL 0.0132 1.0000 0.0132 1491.957 1 20 NA

HL 0.0484 1.0000 0.0484 393.224 1 20 NA

Best candidate to enter: TL

Partial F (enter) = 1491.957

✔ Variable ENTERED: TL

✔ Currently selected variables:

[1] "TL"

>>> FORWARD STEP

F_enter and statistics for all candidate variables:

Var Wilks(full) Wilks(minus) Partial Wilks F df1 df2 Tol(min)

HL 0.0126 0.0132 0.9547 0.902 1 19 0.4768

Best candidate to enter: HL

Partial F (enter) = 0.902

No variable meets the F_enter criterion.

>>> No further changes: stepwise procedure completed.

Final selected variables:

[1] "TL"

## LDA PIPELINE OUTPUT

=============================================

LDA PIPELINE - 42 DPH 70/30 split (stepwise)

=============================================

→ Classes (Sex): F M

→ Equal priors: 0.5 0.5

→ male_lev = M , female_lev = F

▶ Box's M test for homogeneity of covariance matrices:

Box's M not computed: with a single variable only variances can be compared.

▶ LDA model (training sample - 42 DPH 70/30 split (stepwise) )

Call:

lda(form, data = lda_data_G1, prior = priors_equal)

Prior probabilities of groups:

F M

0.5 0.5

Group means:

TL

F 35.93600

M 39.39917

Coefficients of linear discriminants:

LD1

TL 1.063429

▶ Canonical correlations (r):

[1] 0.993364

▶ Global Wilks' Lambda: 0.0132

▶ Stratified bootstrap (1000 iterations) on training sample - 42 DPH 70/30 split (stepwise)

- Mean accuracy (bootstrap, training sample, overall): 99.92 %

- Mean accuracy (bootstrap, training sample, M): 100 %

- Mean accuracy (bootstrap, training sample, F): 99.83 %

- 95% CI (bootstrap, training sample, overall): 100 % - 100 %

- Mean #correct M (bootstrap, training): 12 su 12 [95% CI: 12 – 12 ]

- Mean #correct F (bootstrap, training): 9.98 su 10 [95% CI: 10 – 10 ]

▶ Jackknife cross-validation (LOOCV) on training sample - 42 DPH 70/30 split (stepwise)

- Jackknife accuracy (training sample): 100 %

- Confusion matrix (Jackknife, training sample):

Actual

Predicted F M

F 10 0

M 0 12

▶ Final test on external test sample - 42 DPH 70/30 split (stepwise)

- Test accuracy (external test sample, overall): 100 %

- Confusion matrix (external test sample):

Actual

Predicted F M

F 4 0

M 0 4

▶ Stratified bootstrap (1000 iterations) on external test sample - 42 DPH 70/30 split (stepwise)

- Mean accuracy (bootstrap, external test sample, overall): 100 %

- Mean accuracy (bootstrap, external test sample, M): 100 %

- Mean accuracy (bootstrap, external test sample, F): 100 %

- 95% CI (bootstrap, external test sample, overall): 100 % - 100 %

- Mean #correct M (bootstrap, external test): 4 su 4 [95% CI: 4 – 4 ]

- Mean #correct F (bootstrap, external test): 4 su 4 [95% CI: 4 – 4 ]

## LDA SUMMARY (PAPER-READY, D > 0 = female)

| Parameter | Value |
| --- | --- |
| Discriminant function (paper-ready) | D = -1.063(TL) +40.057 |
| LD1 raw coefficients | TL 1.063 |
| Coefficients used in D (paper-ready) | TL -1.063 |
| Intercept | 40.057 |
| Canonical r (MASS raw / SVD) | 0.99 |
| Canonical r (from Wilks raw) | 0.99 |
| Canonical r (SPSS-like, reported) | 0.89 |
| Wilks' lambda (MASS raw) | 0.013 |
| Wilks' lambda (SPSS-like, reported) | 0.213 |
| p-value (MASS raw) | <0.001 |
| p-value (SPSS-like, reported) | <0.001 |
| Train M (%) | 100 (n=12) |
| Train F (%) | 100 (n=10) |
| Test M (%) | 100 (n=4) |
| Test F (%) | 100 (n=4) |
| Bootstrap train acc (mean, 95% CI) | 99.92% (100.00–100.00%) |
| Bootstrap test acc (mean, 95% CI) | 100.00% (100.00–100.00%) |

### Correct classification (training sample – Jackknife)

- Males: 100.0 % (n = 12)

- Females: 100.0 % (n = 10)

### Correct classification (external test sample)

- Males: 100.0 % (n = 4)

- Females: 100.0 % (n = 4)

**Supplementary figures**


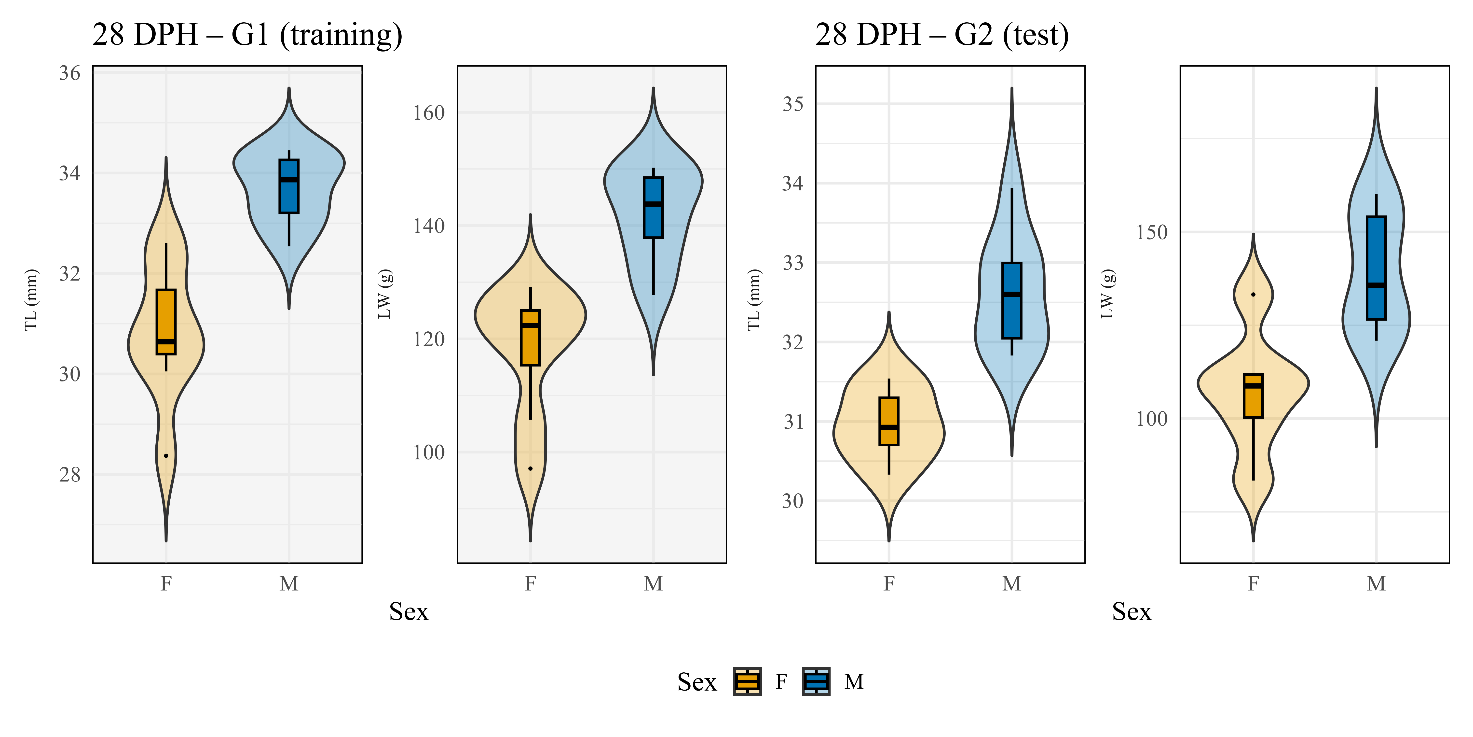


**Supplementary Figure S1. Trait distribution by sex at 28 days post hatching (DPH) in the G1 training group and the G2 external test group.** Violin plots represent the distribution of tarsus length (TL) and live body weight (LW) for males and females under each rearing condition, with violin width proportional to the relative frequency of observations. Embedded boxplots show the median (central line), interquartile range (box), and data dispersion up to 1.5 times the interquartile range (whiskers); individual points indicate outliers when present. Both traits showed consistent sex differences at 28 DPH.


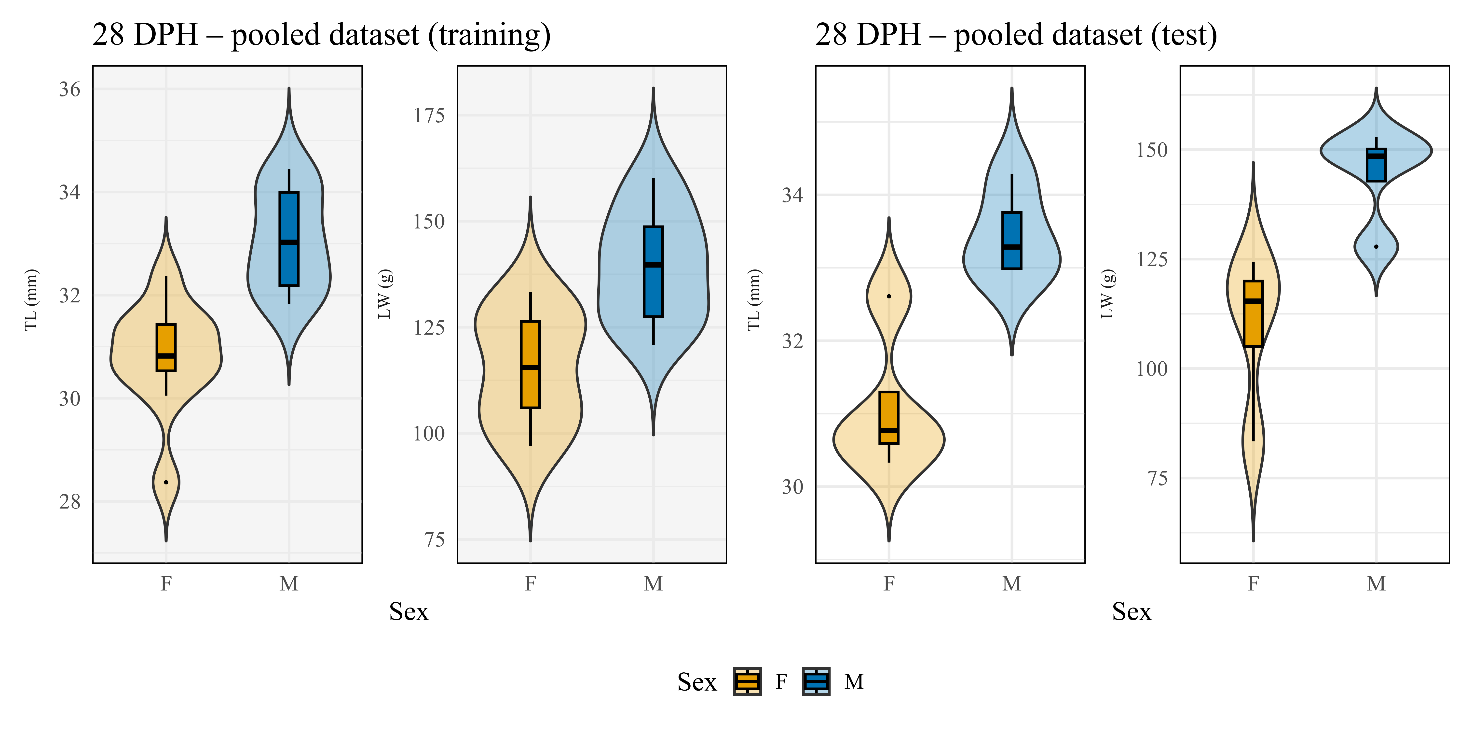


**Supplementary Figure S2. Trait distribution by sex at 28 days post hatching (DPH) in the pooled dataset used for 70/30 training-test split.** Panel A show the 70 % training subset and Panel B the 30 % test subset. Violin plots represent the distribution of tarsus length (TL) and live body weight (LW) for males and females under each rearing condition, with violin width proportional to the relative frequency of observations. Embedded boxplots display the median (central line), interquartile range (box), and data dispersion up to 1.5 times the interquartile range (whiskers); individual points indicate outliers when present. TL and LW were included in the pooled-model discriminant analysis at 28 DPH, yielding moderate but consistent sex differentiation. F: female; M: male


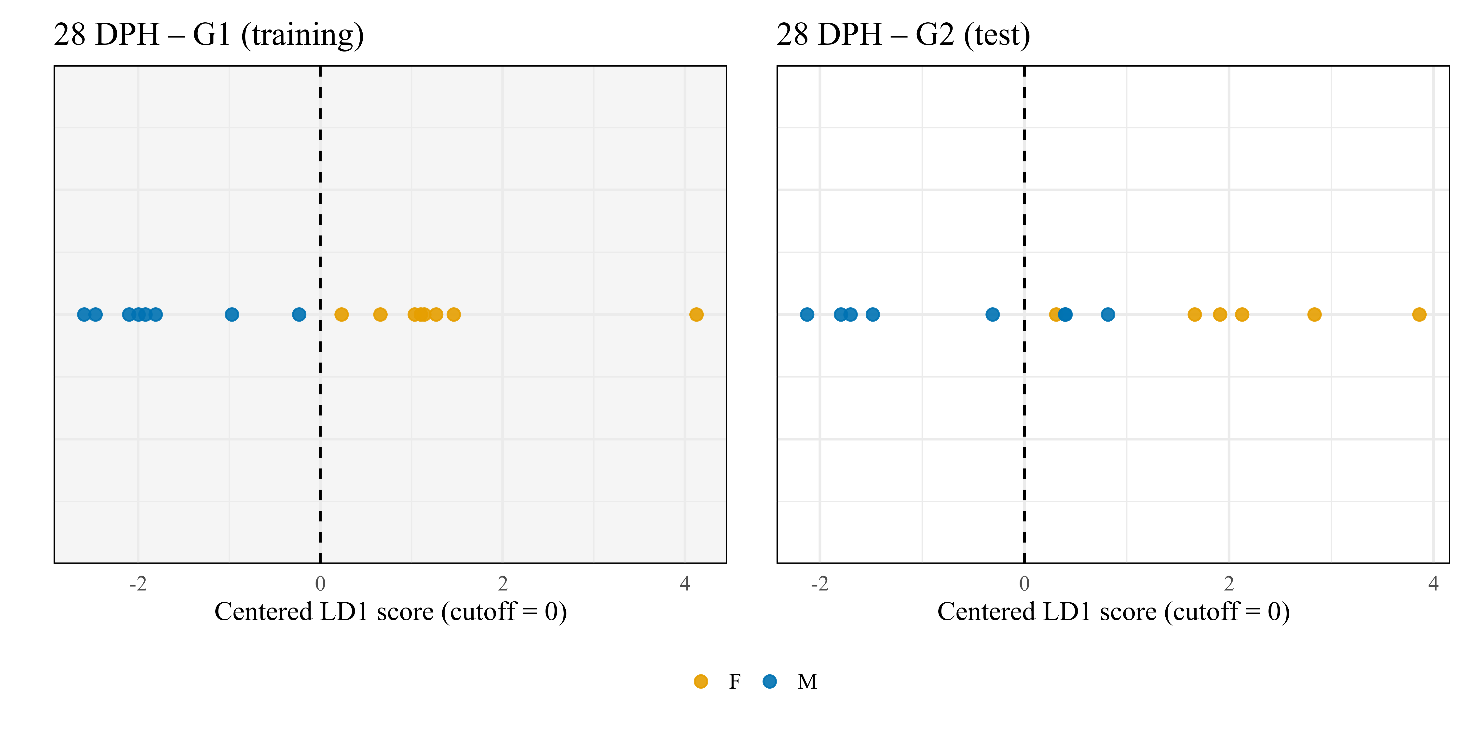


**Supplementary Figure S3. LD discriminant scores by sex at 28 DPH in the G1 training group and the G2 external test group.** Point plot show the centred LD discriminant score for males and females, with the vertical dashed line indicating the classification cutoff (LD = 0). By definition of the oriented discriminant function, values greater than zero (D > 0) correspond to females. F: female; M: male.


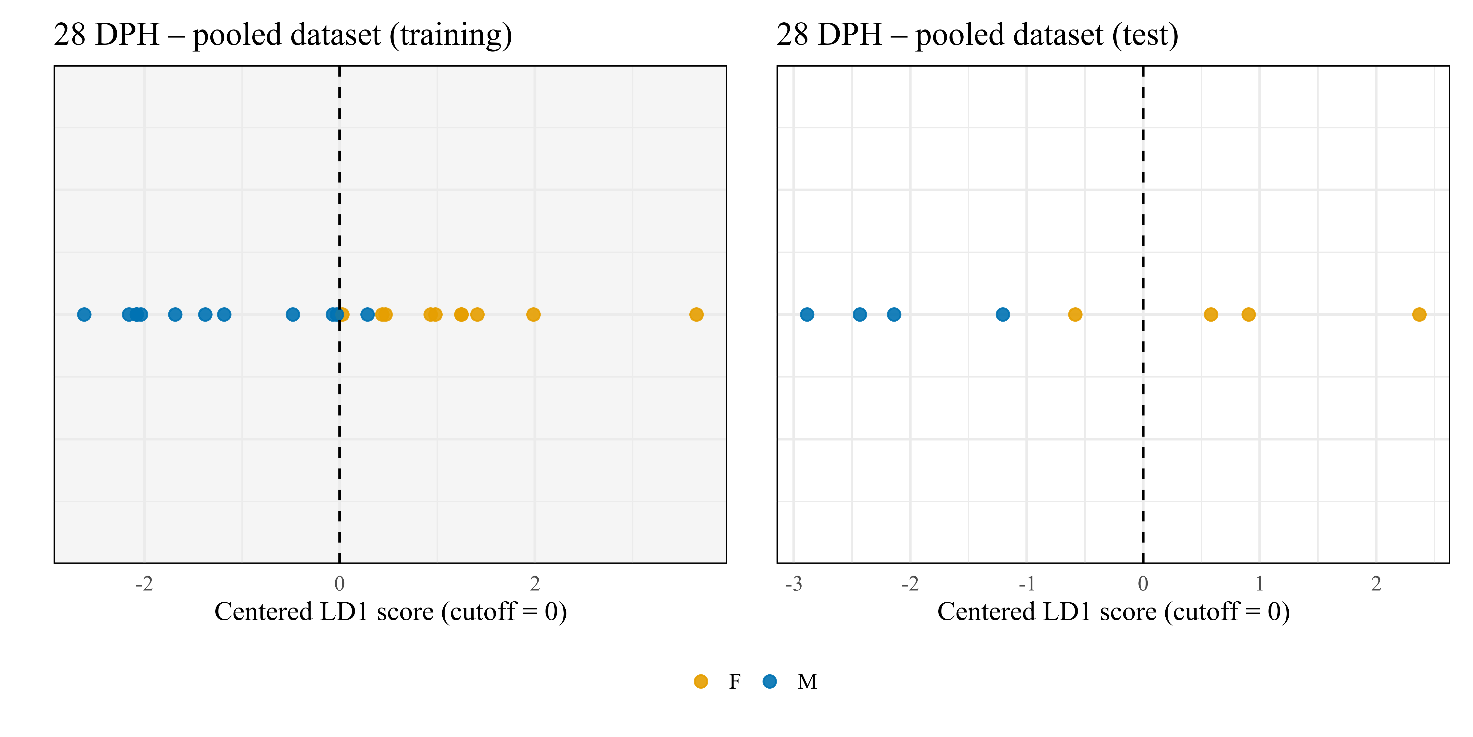


**Supplementary Figure S4. LD discriminant scores by sex at 28 DPH in the pooled dataset used for 70/30 training-test split.** Panel A show the 70 % training subset and Panel B the 30 % test subset. Point represents centred LD discriminant score, with the vertical dashed line marking the classification cutoff (LD = 0). As define in the oriented discriminant function, values greater than zero (D > 0) correspond to females. F: female; M: male.


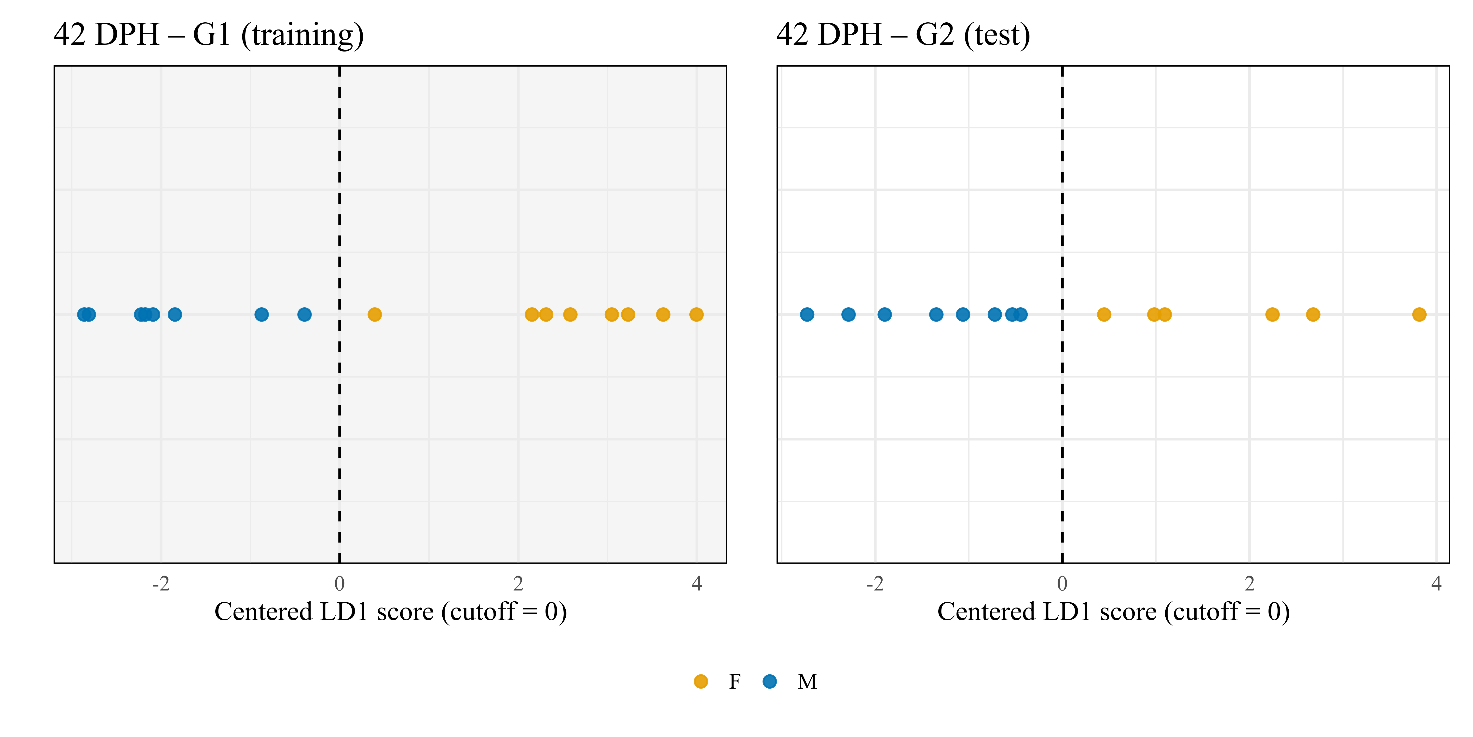


**Supplementary Figure S5. LD discriminant scores by sex at 42 DPH in the G1 training group and the G2 external test group.** Point plot show the centred LD discriminant score for males and females, with the vertical dashed line indicating the classification cutoff (LD = 0). By definition of the oriented discriminant function, values greater than zero (D > 0) correspond to females. F: female; M: male.


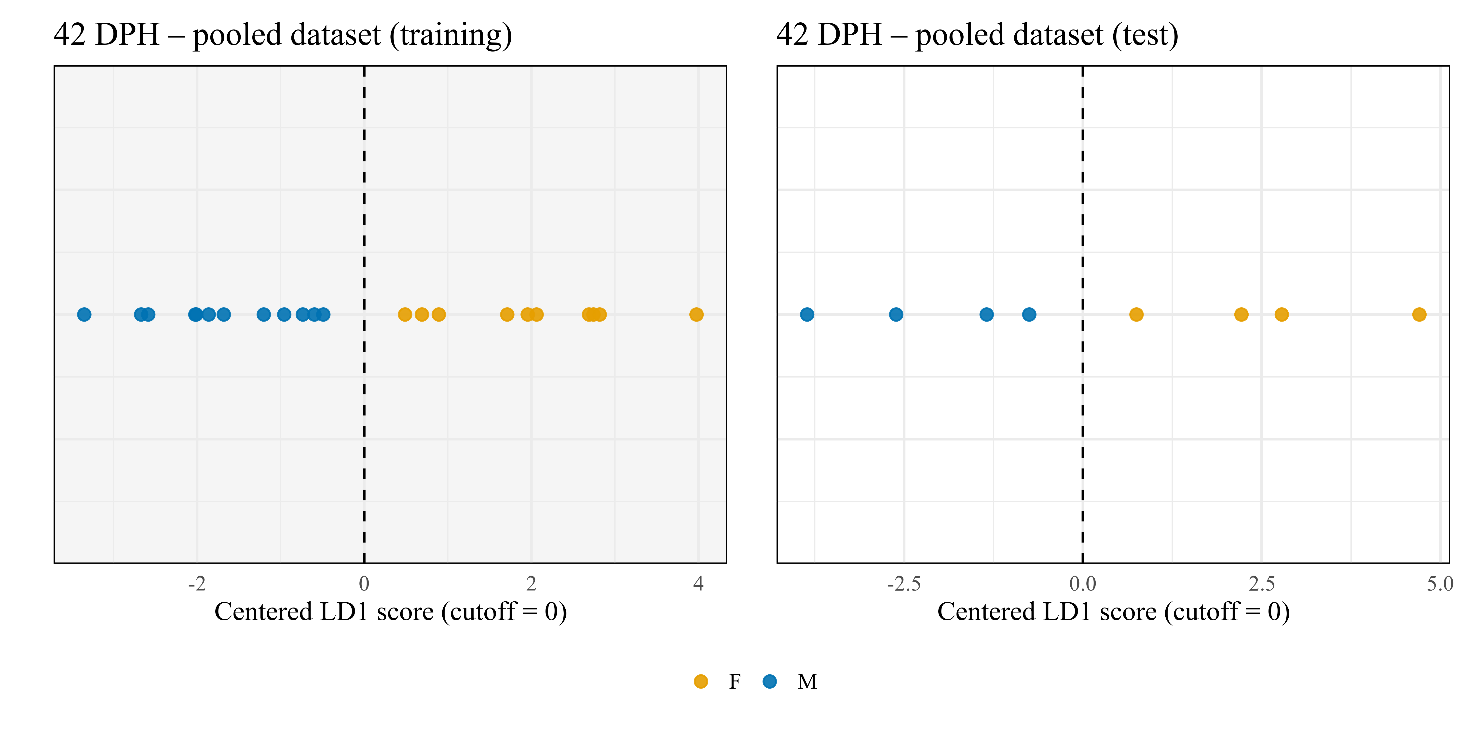


**Supplementary Figure S6. LD discriminant scores by sex at 42 DPH in the pooled dataset used for 70/30 training-test split.** Panel A show the 70 % training subset and Panel B the 30 % test subset. Point represents centred LD discriminant score, with the vertical dashed line marking the classification cutoff (LD = 0). As define in the oriented discriminant function, values greater than zero (D > 0) correspond to females. F: female; M: male.
