# Supplementary material for: Tarsus length as a simple and robust candidate for early sex determination in partridges across contrasting growing contexts: a case study in Rock partridge (Alectoris graeca Meisner, 1804)
Source: Vet Anim Sci. 2026 Apr 12;32:100657. doi: 10.1016/j.vas.2026.100657 (PMC13101291; doi:10.1016/j.vas.2026.100657)
Supplement: Supplementary file 2 [file mmc2.zip › Supplementary Figure Captions.docx]

**Supplementary Figure S1. Trait distribution by sex at 28 days post hatching (DPH) in the G1 training group (intensively reared) and the G2 external test group (wild-like reared).** Violin plots represent the distribution of tarsus length (TL) and live body weight (LW) for males and females under each rearing condition, with violin width proportional to the relative frequency of observations. Embedded boxplots show the median (central line), interquartile range (box), and data dispersion up to 1.5 times the interquartile range (whiskers); individual points indicate outliers (values beyond 1.5 × IQR) when present. Both traits showed consistent sex differences at 28 DPH.

**Supplementary Figure S2. Trait distribution by sex at 28 days post hatching (DPH) in the pooled dataset used for 70/30 training-test split.** Panel A show the 70 % training subset and Panel B the 30 % test subset. Violin plots represent the distribution of tarsus length (TL) and live body weight (LW) for males and females under each rearing condition, with violin width proportional to the relative frequency of observations. Embedded boxplots display the median (central line), interquartile range (box), and data dispersion up to 1.5 times the interquartile range (whiskers); individual points indicate outliers when present. TL and LW were included in the pooled-model discriminant analysis at 28 DPH, yielding moderate but consistent sex differentiation. F: female; M: male

**Supplementary Figure S3. LD discriminant scores by sex at 28 DPH in the G1 training group (intensively reared) and the G2 external test group (wild-like reared).** Point plot show the centred LD discriminant score for males and females, with the vertical dashed line indicating the classification cutoff (LD = 0). By definition of the oriented discriminant function, values greater than zero (D > 0) correspond to females. F: female; M: male.

**Supplementary Figure S4. LD discriminant scores by sex at 28 DPH in the pooled dataset used for 70/30 training-test split.** Panel A show the 70 % training subset and Panel B the 30 % test subset. Point represents centred LD discriminant score, with the vertical dashed line marking the classification cutoff (LD = 0). As define in the oriented discriminant function, values greater than zero (D > 0) correspond to females. F: female; M: male.

**Supplementary Figure S5. LD discriminant scores by sex at 42 DPH in the G1 training group (intensively reared) and the G2 external test group (wild-like reared).** Point plot show the centred LD discriminant score for males and females, with the vertical dashed line indicating the classification cutoff (LD = 0). By definition of the oriented discriminant function, values greater than zero (D > 0) correspond to females. F: female; M: male.

**Supplementary Figure S6. LD discriminant scores by sex at 42 DPH in the pooled dataset used for 70/30 training-test split.** Panel A show the 70 % training subset and Panel B the 30 % test subset. Point represents centred LD discriminant score, with the vertical dashed line marking the classification cutoff (LD = 0). As define in the oriented discriminant function, values greater than zero (D > 0) correspond to females. F: female; M: male.
