# Supplementary figures and images for: Tarsus length as a simple and robust candidate for early sex determination in partridges across contrasting growing contexts: a case study in Rock partridge (Alectoris graeca Meisner, 1804)
Source: Vet Anim Sci. 2026 Apr 12;32:100657. doi: 10.1016/j.vas.2026.100657 (PMC13101291; doi:10.1016/j.vas.2026.100657)

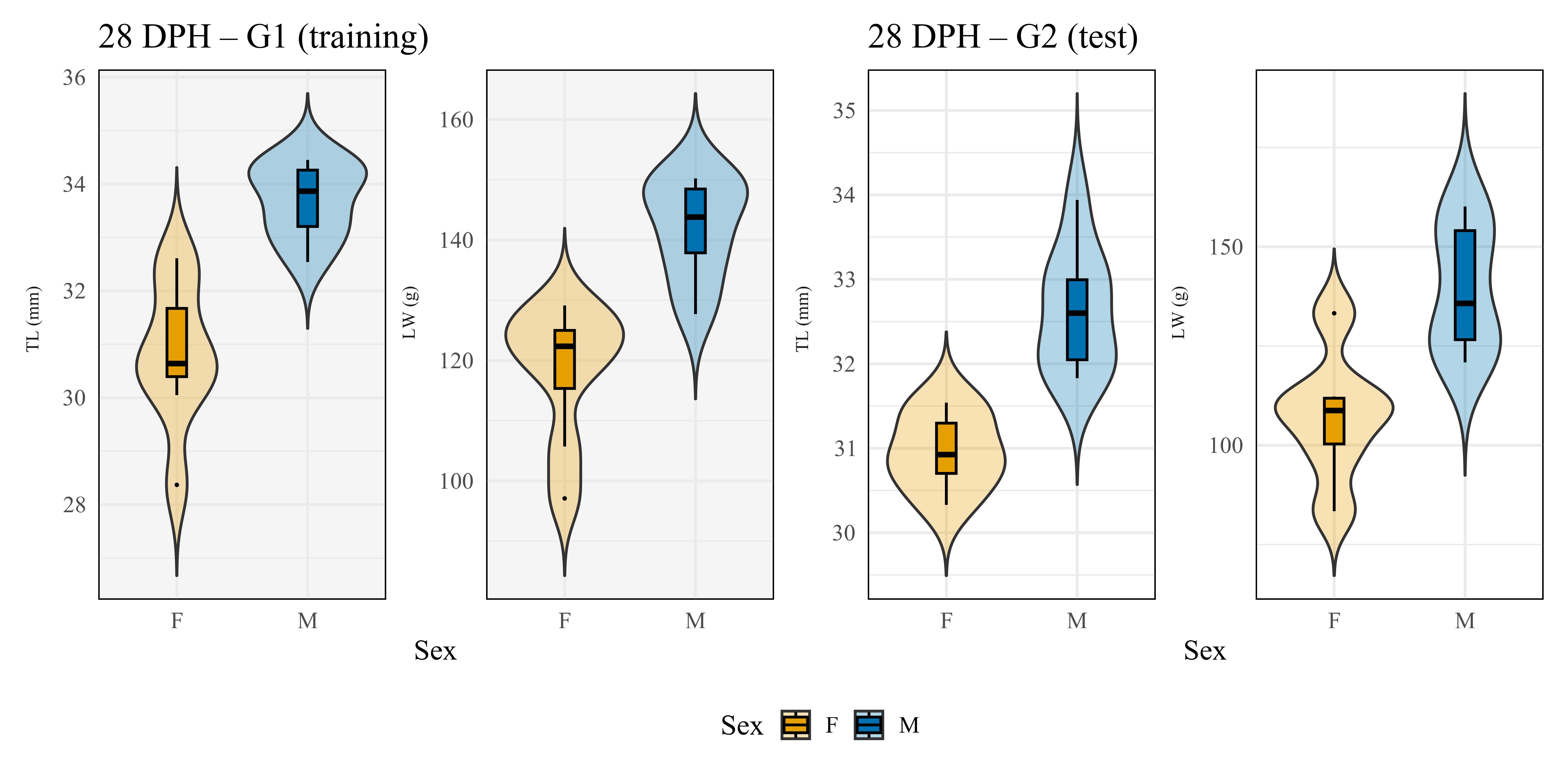

Supplement: Supplementary file 2 [file mmc2.zip › Supplementary_Figure_S1.tiff]

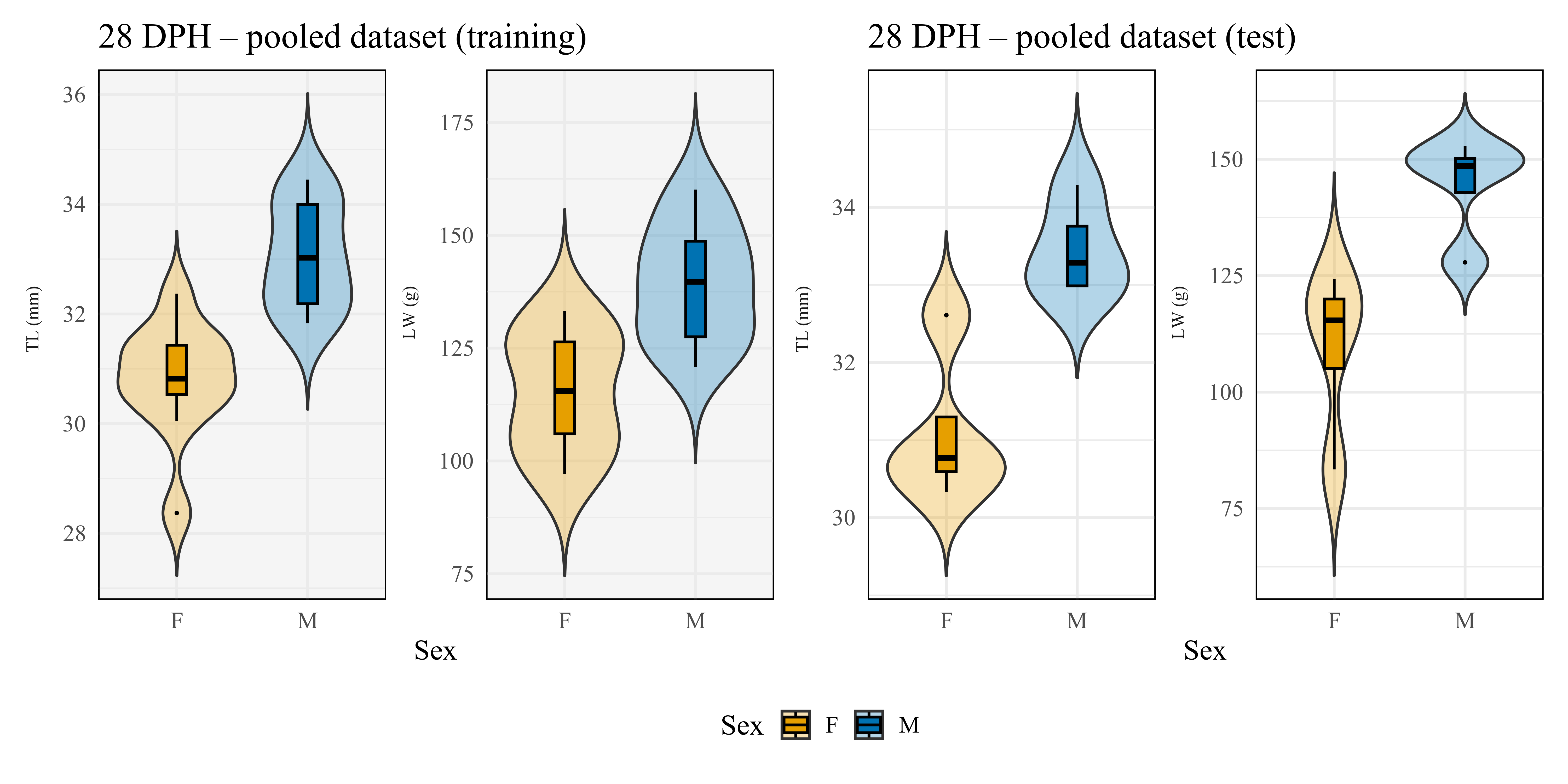

Supplement: Supplementary file 2 [file mmc2.zip › Supplementary_Figure_S2.tiff]

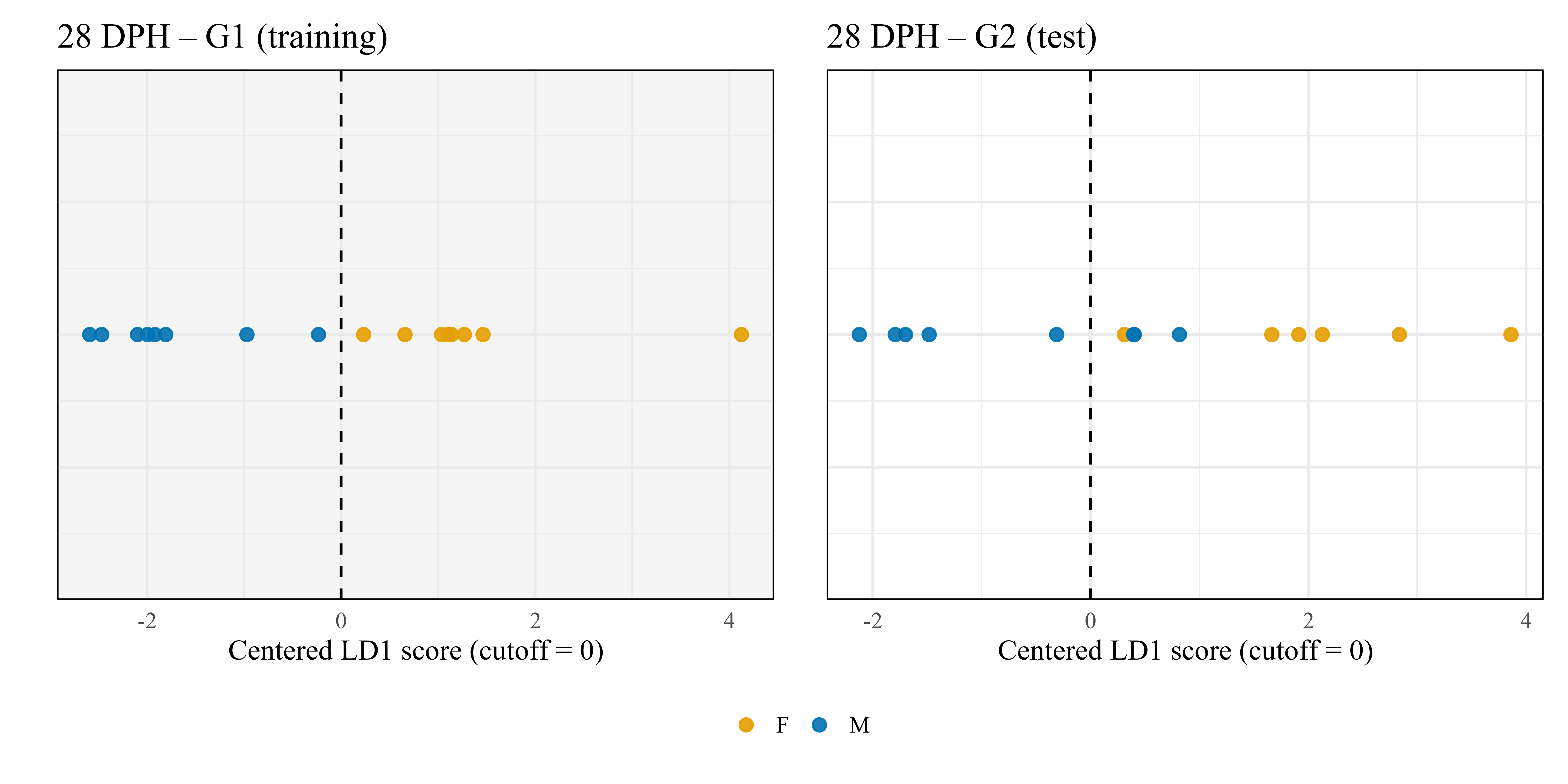

Supplement: Supplementary file 2 [file mmc2.zip › Supplementary_Figure_S3.tiff]

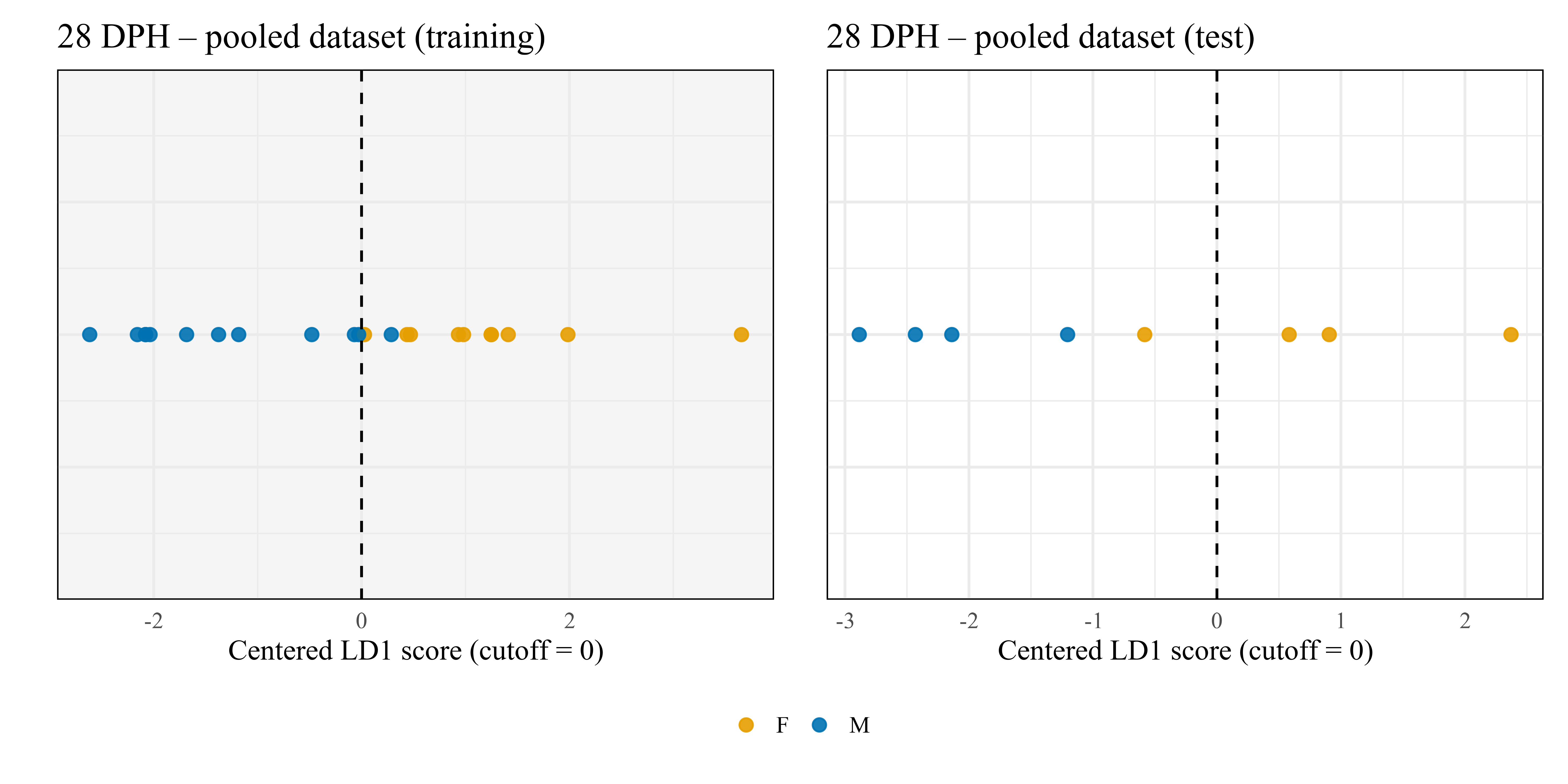

Supplement: Supplementary file 2 [file mmc2.zip › Supplementary_Figure_S4.tiff]

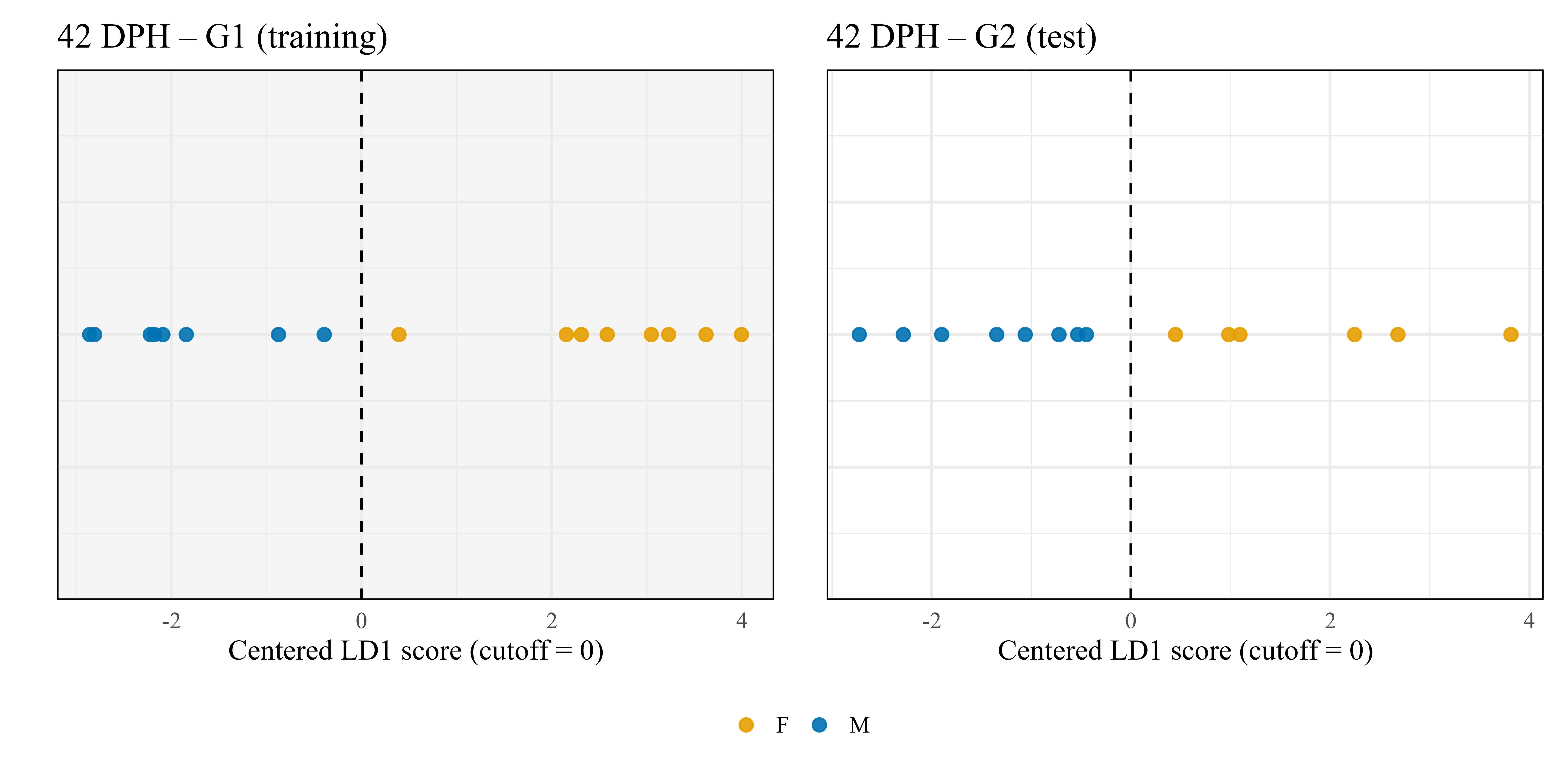

Supplement: Supplementary file 2 [file mmc2.zip › Supplementary_Figure_S5.tiff]

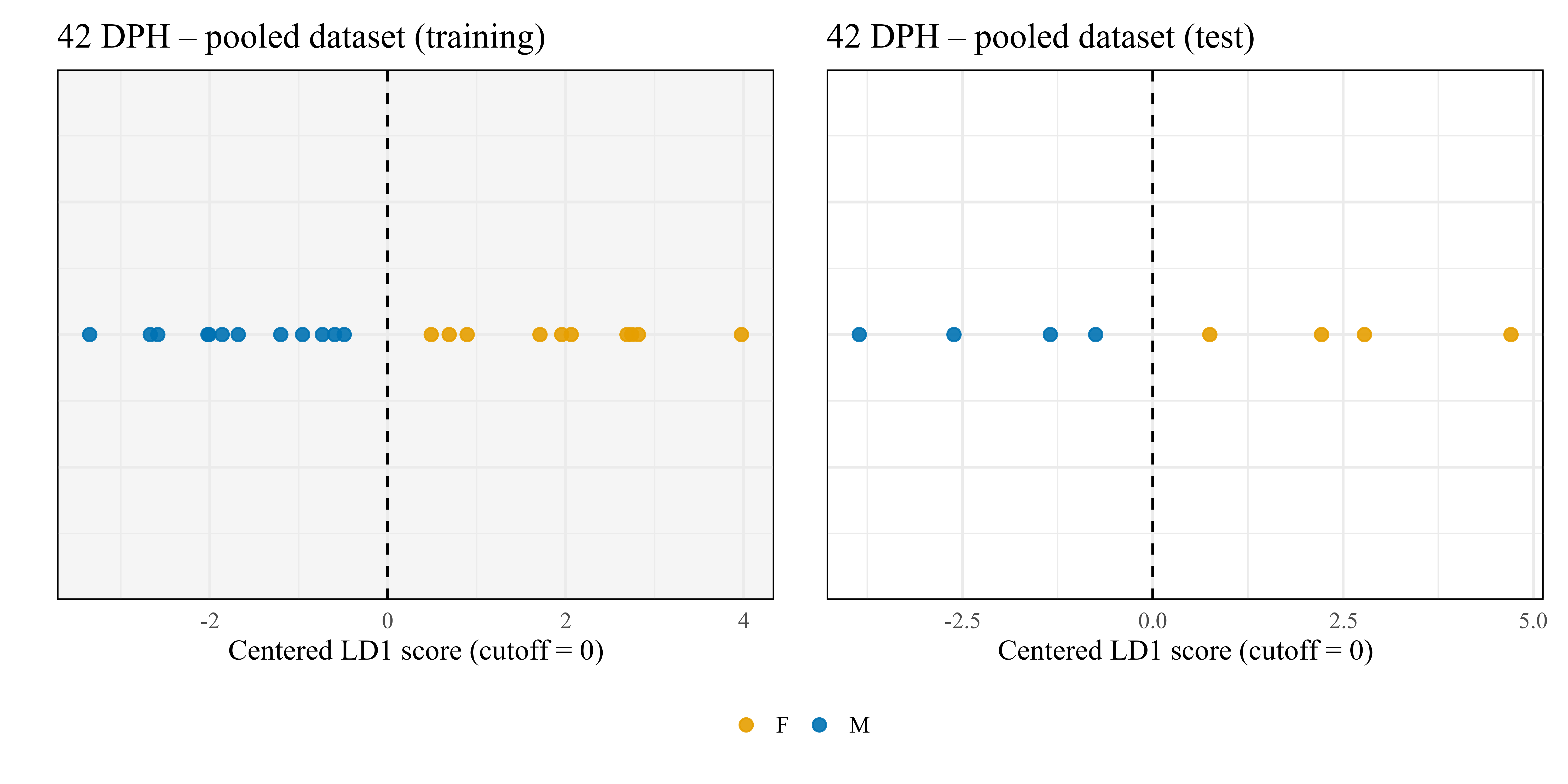

Supplement: Supplementary file 2 [file mmc2.zip › Supplementary_Figure_S6.tiff]
